# *In vitro* and *in silico* determination of glutaminyl cyclase inhibitors†

**DOI:** 10.1039/c9ra05763c

**Published:** 2019-09-19

**Authors:** Phuong-Thao Tran, Van-Hai Hoang, Jeewoo Lee, Tran Thi Thu Hien, Nguyen Thanh Tung, Son Tung Ngo

**Affiliations:** Department of Pharmaceutical Chemistry, Hanoi University of Pharmacy Hanoi Vietnam thaotp119@gmail.com; Institute of Research and Development, Duy Tan University Da Nang 550000 Vietnam; Laboratory of Medicinal Chemistry, College of Pharmacy, Seoul National University Seoul Korea; Vietnam University of Traditional Medicine Hanoi Vietnam; Institute of Materials Science, Vietnam Academy of Science and Technology Hanoi Vietnam; Laboratory of Theoretical and Computational Biophysics, Ton Duc Thang University Ho Chi Minh City Vietnam ngosontung@tdtu.edu.vn; Faculty of Applied Sciences, Ton Duc Thang University Ho Chi Minh City Vietnam

## Abstract

Alzheimer's disease (AD) is the most common form of neurodegenerative disease currently. It is widely accepted that AD is characterized by the self-assembly of amyloid beta (Aβ) peptides. The human glutaminyl cyclase (hQC) enzyme is characterized by association with Aβ peptide generation. The development of hQC inhibitors could prevent the self-aggregation of Aβ peptides, resulting in impeding AD. Utilizing structural knowledge of the hQC substrates and known hQC inhibitors, new heterocyclic and peptidomimetic derivatives were synthesized and were able to inhibit the hQC enzyme. The inhibiting abilities of these compounds were evaluated using a fluorometric assay. The binding mechanism at the atomic level was estimated using molecular docking, free energy perturbation, and quantum chemical calculation methods. The predicted log(BBB) and human intestinal absorption values indicated that these compounds are able to permeate the blood–brain barrier and be well-absorbed through the gastrointestinal tract. Overall, 5,6-dimethoxy-*N*-(3-(5-methyl-1*H*-imidazol-1-yl)propyl)-1*H*-benzo[*d*]imidazol-2-amine (1_2) was indicated as a potential drug for AD treatment.

## Abstract 2

Rational design of new hQC inhibitors.

## Graphical abstract

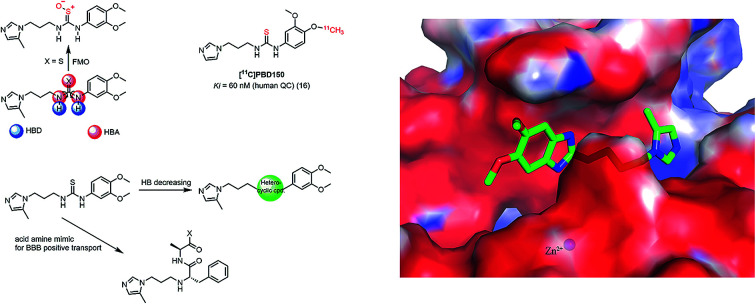

## Introduction

Alzheimer's disease (AD) is known to be one of the most critical types of dementia, affecting several million people worldwide. There are *ca.* 10 million patients arising annually.^1^ The financial cost of treatment and Medicare for AD patients is rising rapidly.^1^ AD is strongly associated with the self-aggregation of amyloid beta (Aβ) peptides,^2,3^ which are a heterogeneous mixture of peptides having different solubility, stability, and biological and toxic properties.^3,4^ These peptides are generated from the single-pass transmembrane amyloid precursor protein (APP) by several proteases at different sites. Several species of Aβ peptides have been observed, whereas Aβ_42_ and Aβ_40_ form the majority.^2^ The self-assembly of Aβ peptides produces several products including random coils, oligomers, photofibrils, fibrils, and plaques.^5–8^ This process results in the impairment of memory function and the loss of neurons and leads to synaptic dysfunction.^9–11^

Pyroglutamate Aβ peptides (AβpE) were found in the self-aggregation of Aβ peptides and act as initiators for Aβ accumulation. In fact, AβpE are only found in AD brains and constitute approximately 50% of the total Aβ.^12^ Their formation is a multistep process requiring the loss of two or ten amino acids to expose the *N*-terminal glutamate at the third or eleventh position, followed by intramolecular dehydration of the exposed glutamate. The Aβ formation process results in the loss of three or six charges for Aβ3pE or Aβ11pE, respectively, leading not only to higher hydrophobicity but also to more rapid forming of β-sheet structures and, thus, greater stability and aggregation propensity of the Aβ*x*pE. Moreover, newly formed lactam rings are more stable under aminopeptidase medium than is full length Aβ.^13^ All of these factors result in AβpE acting as a seed species for toxic Aβ structural aggregation in the early stage of AD.^14^

**Fig. 1:**
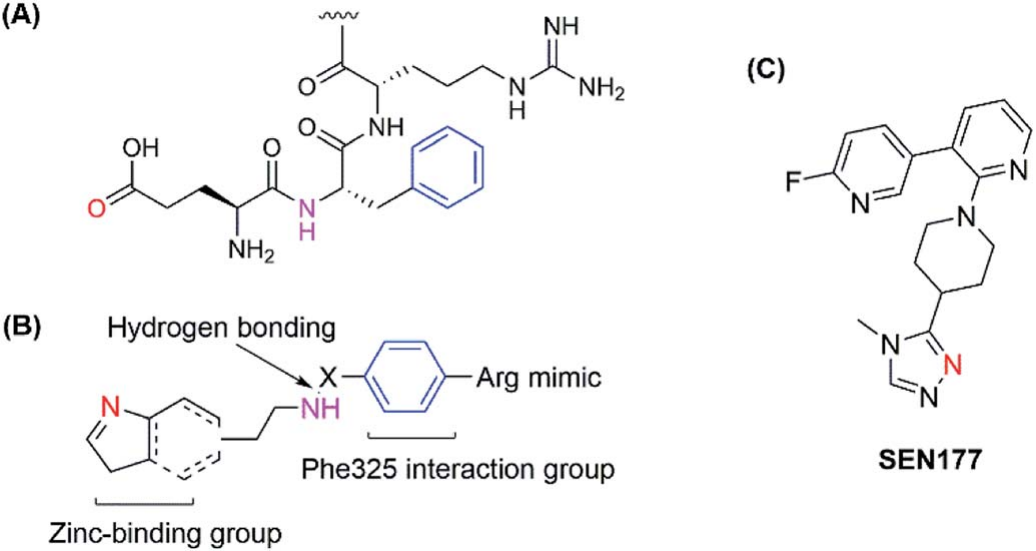
(A) Structure of the hQC substrate; (B) hQC inhibitor scaffold for the 1^st^ pocket; (C) structure of SEN177.

The human glutaminyl cyclase (hQC) is a zinc-dependent enzyme that catalyzes the intramolecular cyclization of the *N*-terminal glutamine residue into pyroglutamic acid-liberating ammoniac. It is a key enzyme for some hormones post-translation, protecting them from proteolytic degradation.^15,16^ It also converts *N*-truncated Aβ into AβpE, a more toxic Aβ species.^17^ Therefore, hQC is regarded as an initiator agent for pathological Aβ accumulation, and inactive hQC can prevent and treat AD. Some kinds of hQC inhibitors were discovered with imidazole, triazole or benzimidazole as zinc-binding motifs. Most of these inhibitors mimicked two or three amino acids of the sequence of *N*-truncated Aβ, NH_2_–Glu–Phe–Arg. They all contain zinc-binding group, a hydrogen-bonding donor and an aromatic group to interact with Phe325 in the pocket as a critical amino acid for potent binding.^18–22^ Recently, a new binding pocket was reported with an orientation opposite to that of the Phe325 pocket, based on the discovery of SEN177. Crystal studies of the SEN177–hQC complex showed two interactions between the enzyme and the inhibitor in terms of inhibitor orientation: the pyridine ring with Trp207 and the fluorine atom of 2-fluoropyridine with the hydrogen atom of His330 (ref. 23) (Fig. 1). However, up to now, only PQ-912 is being studied in clinical trials and has completed a phase IIa trial.^24^

**Fig. 2:**
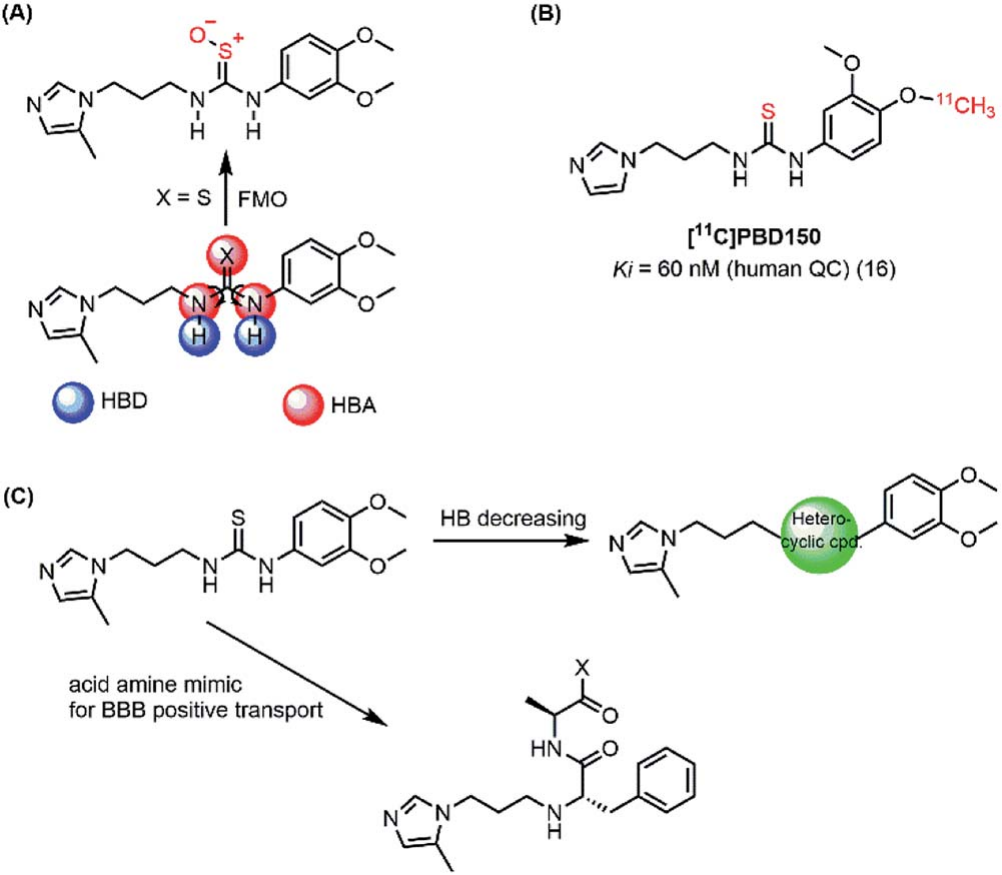
(A) Urea/thiourea property issues and phase I metabolism restrict BBB penetration; (B) structure of [^11^C]PBD150; (C) diagram of design new hQC inhibitors.

In the present work, both computational and experimental studies were performed to design two new potential inhibitory hQC series based on structural knowledge of the hQC substrates and known hQC inhibitors.^19–22,25^ To the best of our knowledge, most potential hQC inhibitors contain substituted urea or thiourea scaffolds. The potency of thiourea derivatives are greater than that of corresponding ureas.^18^ Both thiourea and urea types contribute not only two hydrogen bonding donors (HBD) and three hydrogen bonding acceptors (HBA) but also flexible bonding (Fig. 2A). It looks like that these properties cause blood–brain barrier (BBB) penetration issue based on Hassan Pajouhesh's suggestion for CNS drug design and discovery.^26^ Moreover, thiourea group is unstable, it can be metabolized by flavin monooxygenase (FMO) to reactive species which can alkylate proteins and nucleic acids.^27^ Therefore, they are limited in their ability to penetrate through BBB and in *in vitro*–*in vivo* correlation. In addition, compound [^11^C]PDB150, the thiourea-containing hQC inhibitor with the potential inhibitory efficacy, *K*_i_ value of 60 nM (16), was determined to impossible permeation the BBB (Fig. 2B).^28^ After these considerations, we designed the new hQC inhibitory series without urea and thiourea scaffolds. In the first series, thiourea groups were replaced by a bioisostere or a fused heterocyclic ring. In particular, we also study the effect of rigid structures on the region of inhibitor–enzyme interaction. The second series was designed based on urea/thiourea scaffold removal combining two amino acid residues of the terminal substrate. Be contained a necessary nutrient for the brain and amino acid that they would be present in brain fluid through positive transport by protein pumps (Fig. 2C). The hQC inhibiting abilities of these compounds were determined using a fluorometric assay with a fluorogenic substrate. The binding mechanisms of the imidazole derivatives to the hQC enzyme were then evaluated using a combination of molecular docking, alchemical free energy, and quantum chemical calculations. In particular, molecular docking was employed to predict binding poses between the trial inhibitors and hQC. The binding process was then refined using conventional molecular dynamics (MD) simulations, and the physical insights provided by these processes were clarified using the free energy perturbation (FEP) method. The influence of Zn^2+^ to the ligand-binding affinity was characterized through quantum chemical calculation. The good agreement between the computations and experiments indicates that 5,6-dimethoxy-*N*-(3-(5-methyl-1*H*-imidazol-1-yl)propyl)-1*H*-benzo[*d*]imidazol-2-amine (1_2) is able to be used as a drug with high potential in preventing AD.

## Materials and methods

### Synthetic experiments

All chemical reagents were commercially available and used directly without any purification. Silica gel column chromatography was performed on silica gel 60 of 230–400 mesh from Merck. ^1^H NMR spectra were recorded on a JEOL JNM-LA 300 at 300 MHz by Bruker Analytik or on a DE/AVANCE Digital 400 at 400 MHz by Bruker Analytik, with Me_4_Si as a reference standard. Mass spectra were recorded on a VG Trio-2 GC-MS instrument and a 6460 Triple Quad LC-MS instrument. All final compounds were assessed for purity by high performance liquid chromatography (HPLC) on Agilent 1120 Compact LC (G4288A) system *via* the following conditions. Column: Agilent TC-C18 column (4.6 mm × 250 mm, 5 μm). Mobile phase A: 0.1% TFA in MeOH, mobile phase B: 0.1% TFA in water (v/v) in 30 min. Wavelength: 254 nM. Flow: 0.7 to 1.0 mL min^−1^. According to the HPLC analyses, all final compounds showed a purity of ≥95%. In addition, detailed information of inhibitors was described in the supplementary (ESI file†).

### Bioactive assay

The synthesized compounds were evaluated for hQC inhibition by a fluorometric assay with a fluorogenic substrate, Glu-AMC·HBr (l-glutamine 7-amido-4-methylcoumarin, BACHEM, Switzerland), and an auxiliary enzyme, pyroglutamyl peptidase (pGAPase, 50 units, Qiagen, Germany), as our previously reported.^29^ The used buffer consisted of 25 mM HEPES (Sigma), with pH 7.0, adjusted with HCl. The reaction mixture contained 25 μL of substrate (0.4 mM), 50 μL of the test compound (0.016, 0.08, 0.4 and 2 μM), 25 μL of pGAPase (0.2 unit). After incubation in 96-well black plates (Greiner, Austria) for 10 min at 37 °C, the reaction was started by adding 50 μL of hQC solution (0.04 μg mL^−1^). The excitation/emission wavelengths at 380/460 nm were correlated with the amount of ACM, the last product of the enzymatic process.

### Initial shape of glutaminyl cyclase and inhibitors

The structure of hQC was obtained from the Protein Data Bank with ID: 3PBB^30^ referring to the previous work.^31^ The 3D-structural inhibitors were built utilizing Gabedit package,^32^ and these structures were optimized in the gas phase using quantum chemical calculations with hybrid functional B3LYP at the 6-31G(d,p) level of the basic set.

### Molecular docking

The binding poses and affinities trials of inhibitors for the hQC enzyme were estimated using Autodock4.2 packages.^33^ In particular, the input file for the molecular docking simulations was prepared using AutodockTools 1.5.6.^33^ The docking grid was chosen as the center of the hQC active site, with the size of 60 × 60 × 60 and the spacing of 0.375 Å. The genetic algorithm (GA) run was chosen as 50, with the population size set to 300. The GA number of evaluations was set as 25 000 000, with the number of generations at 27 000. The Zn^2+^ atom was also involved during the docking simulation as mention in the Fig. 4.

### Atomistic molecular dynamics simulation

The hQC protein including Zn^2+^ ion was parameterized using the AMBER99SB-ILDN force field.^34^ In particular, the active site of hQC including Zn(ii) (Fig. S1 and S2†) was parameterized by using a Python Base Metal Center Parameter Builder (MCPB.py) package.^35^ The force constant and detail of coordination link between Zn(ii) and its ligands were determined as described in Fig. S1 and Table S2.† The atomic charges was estimated *via* the restrained electrostatic potential (RESP) method.^36^ The RESP calculation was carried out by using quantum chemical calculation using B3LYP functional at 6-31G(d) level of basic set in gas phase (Fig. S2†). The protonation state of hQC enzyme was predicted *via* H++ server with pH condition was selected at 7.4.^37^ Moreover, binding poses between inhibitors and the hQC enzyme were generated using Autodock4.2 (ref. 33) as mentioned above. The inhibitors were represented by employing the general amber force field (GAFF),^38^ wherein the atomic charges were assigned by the restrained electrostatic potential (RESP) method through quantum chemical calculations using the MP2 functional at the 6-31G(d,p) level of the basic set.^36^ The complexes were put into a dodecahedron periodic boundary condition box with the volume of ∼497.30 nm^3^. Water molecules were represented using the TIP3P water model.^39^ Na^+^ ions were added to neutralize the soluble system.

The simulation parameters were obtained by referring to previous publications.^40^ In particular, GROMACS 5.1.3 (ref. 41) with GPGPU (general-purpose computing on graphics processing units) acceleration was employed to simulate the complexed hQC-ligand in solution. The non-covalent bond pair cut-off was of 0.9 nm. The electrostatic potential was treated by employing the fast smooth particle-mesh Ewald electrostatic method with a cut-off of 0.9 nm.^42^ The effect of van der Waals (vdW) interactions of each pair was in the range of 0.9 nm. The first step of the simulation was energy minimization through the steepest descent method. Then, 500 ps of positional restraint simulation in NVT and NPT ensembles followed. A conventional MD simulation with a length of 50 ns was carried out to relax the complex to the stable state. The system coordinates were monitored every 10 ps. There were 3 independent MD trajectories performed. The last snapshots of the conventional MD simulations were used in the initial free energy calculation with the FEP method.^43^

### Free energy calculation

Free energy calculations were carried out using the FEP method,^43^ since it is one of the most accurate methods to date.^44^ The double annihilation-binding free energy method was performed to determine the binding free energy between two biomolecules, referring to a previous study.^45^ 16 values of the coupling parameter *λ* were employed to modify the Hamiltonian over the computations. In particular, *λ* was chosen as 0.00, 0.10, 0.20, 0.35, 0.50, 0.65, 0.80, and 1.00 to change the electrostatic interactions, and *λ* was chosen as 0.00, 0.10, 0.25, 0.35, 0.50, 0.65, 0.75, 0.90, and 1.00 referring the previous study.^44^ The total free energy change during the Hamiltonian alteration was calculated using Bennet's acceptance ratio method.^46^ The difference between the free energy changes during two processes was determined by demolishing the ligand from the solvated complex and isolating the systems corresponding to the absolute binding free energy of the ligand to the enzyme.

### Structural analysis

The surface charge distribution of proteins was estimated using the Adaptive Poisson–Boltzmann Solver (APBS) package.^47^ The sidechain (SC) contact was computed when the distances between non-hydrogen atoms were smaller than 0.45 nm. The hydrogen bond (HB) was calculated when the distance between the donor (D) and acceptor (A) was smaller than 0.35 nm and the angle between A–H–D was larger than 135° according to the previous work.^48^ It should be noted that H indicates a hydrogen atom.

### Estimated physicochemical properties, mPO score, log(BBB) and HIA values

The open bioactivity prediction online-site Molinspiration (https://www.molinspiration.com/cgi-bin/properties) was used for the topological polar surface area (TPSA) prediction. log *P* and log *D* were predicted using Medchem Designer 5.5 Simulation Plus. The Online-site Chemicalize of ChemAxon was used for p*K*_a_ prediction (https://chemicalize.com/#/calculation). The CNS mPO was calculated followed the previously published method.^49^ The blood–brain barrier (BBB) crossing ability of a ligand was predicted using the PreADME protocol,^50^ referring previous studies.^40,51^ The human intestinal absorption capacity of a ligand was also estimated using the same application.^50^

## Results and discussion

### Chemistry

**Scheme 1:**
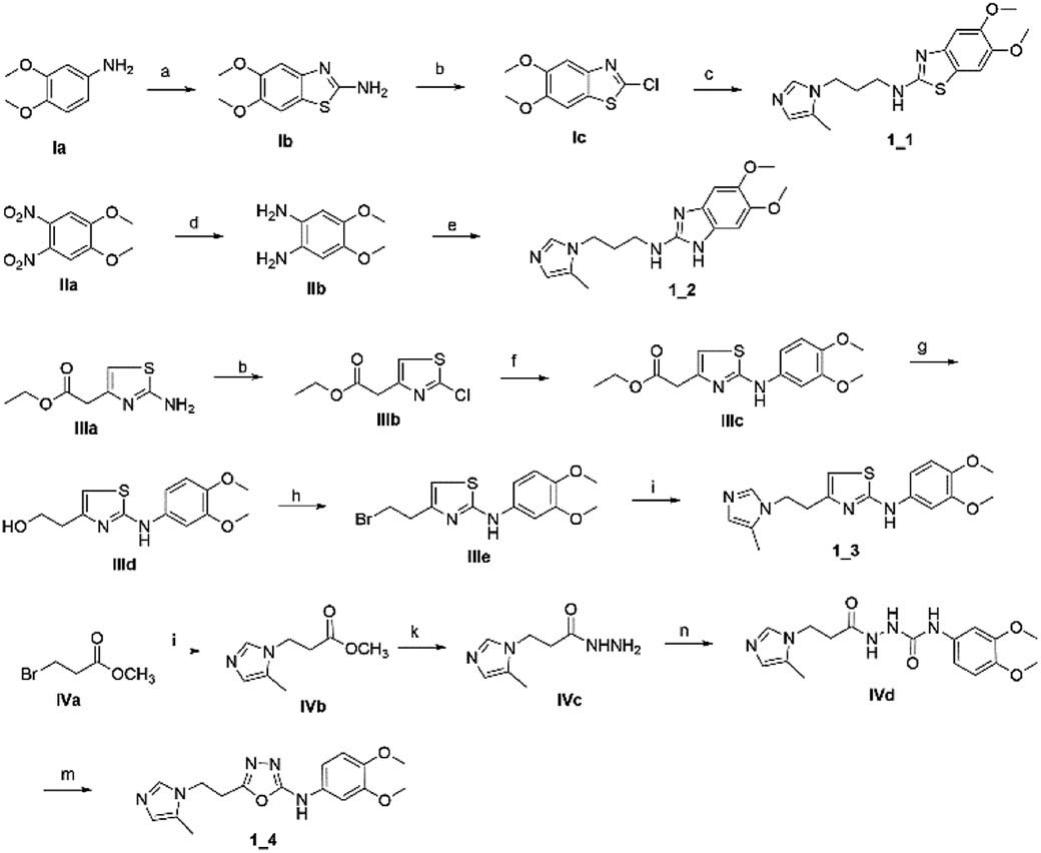
Synthetic scheme for 1_x series compounds. Conditions and reagents: (a) KSCN, AcOH, Br_2_, 0 °C to r.t., 12 h; (b) isoamyl nitrate, CuCl_2_, MeCN, 0 °C to r.t., 12 h; (c) Pd(OAc)_2_, P(Cy)_3_, NaO*t*-Bu, DMA, 100 °C, 12 h; (d) H_2_, Pd/C, MeOH/THF, r.t.; (e) 1-(3-isothiocyanatopropyl)-5-methyl-1*H*-imidazole, THF/DMF (1/1), r.t., 2 h then EDC.HCl, 70 °C, 1 h; (f) *p*-TSA, EtOH, reflux, o.n.; (g) LAH, THF, 0 °C to r.t., 1 h; (h) CBr_4_, Ph_3_P, THF, r.t., 2 h; (i) 5-methyl-1-trityl-1*H*-imidazole, MeCN, reflux, 24 h then MeOH, THF, reflux, o.n.; (k) N_2_H_4_·H_2_O, EtOH, r.t., o.n.; (n) 3,4-dimethoxyaniline, CDI, DCM-DMF, r.t., o.n.; (m) PBr_3_, reflux, 2 h.

To synthesize trial compounds for our study, we prepared 1-(3-isothiocyanatopropyl)-5-methyl-1*H*-imidazole following the reported literature^19^ (Scheme 3 of the ESI file†) to couple with other intermediates as described in Scheme 1. Specifically, 2-aminobenzothiazole Ib was prepared from 3,4-dimethoxyaniline under acidic conditions and using the active reagent bromine. Then, the amino group of 2-aminobenzothiazole was converted to chloride Ic under a Sandmeyer-like reaction for use in the last step, which was a Buchwald–Hartwing reaction to provide compound 1_1. Next, the diamino derivative IIb was introduced thiocyanate to form an *o*-amino thiourea intermediate, which was cyclized under EDC conditions to afford 1_2.^10,11^ In the case of the unfused thiazole ring 1_3, the amino group of IIIa was also converted to chloride IIIb under diazonium conditions for aromatic nucleophilic substitution with Ia to yield intermediate IIIc. This compound was reduced under LAH, and Apple conditions were applied to generate a bromide derivative, which was coupled with trityl protected 4(5)-imidazole to provide 1_3. The last compound 1_4, a 2-amino oxadiazole ring, was produced from the hydrazide carboxamide intermediate IVd under dehydrated cyclization conditions.

**Scheme 2:**
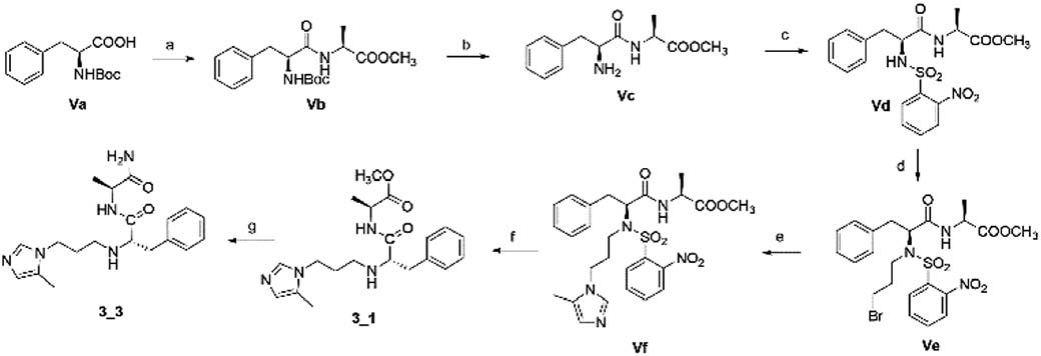
Synthetic route for 3_x series compounds. Conditions and reagents: l-alanine methyl ester hydrochloride, HOBt, EDC, TEA, MC, r.t., 12 h; (b) TFA, TIPS, DMC, r.t., o.n.; (c) 2-nitrobenzenesulfonyl chloride, TEA, DMC, 0 °C to r.t., 4 h; (d) 1,3-dibromopropane, Cs_2_CO_3_, DMF, 60 °C, 6 h; (e) 5-methyl-1-trityl-1*H*-imidazole, MeCN, reflux, 24 h then MeOH, THF, reflux, o.n.; (f) thiophenol, K_2_CO_3_, MeCN, r.t., o.n.; (g) NH_3_, MeOH, r.t., o.n.

The second series, peptidomimetic, was started with 2 amino acids, phenylalanine Va and alanine, forming the new peptide bond Vb (Scheme 2). The protected amino group Vb was exposed to free amine Vc under acidic conditions for *N*-alkylation, which was activated prior by a sulfonyl group, to obtain the intermediate Ve.^12^ Next, an imidazole group was introduced for Ve, followed by removing the sulfonyl group through strong nucleophilic substitution to afford the final compound 3_1. Last, compound 3_3 was prepared from 3_1 under ammonium conditions.

### Biological activity

As mentioned above, the inhibiting abilities of the trial compounds toward the hQC protein were estimated using a binding assay, referring the previous study.^29^ Obtained results are described in Table 1. Compounds with extremely low half-maximal inhibitory concentrations (IC_50_ > 10 000 nM) are not shown in Table 1 for clarity, since these compounds insignificantly influenced the hQC enzyme. All six compounds showing in Table 1 emerge as potential inhibitors of the hQC protein, with IC_50_ values ranging from 0.64 to 0.11 μM. However, when comparison to reference thiourea compound 1,^19^ all of six compounds showed a drop of potency from 3.7 to 22 times, even though, they contained the similar structure to thiourea motifs, such as: benzothiazole (1_1), benzimidazole (1_2), or the bioisosteres: 2-aminothiazole (1_3) and 2-amino oxadiazole (1_4). As the previous study on SAR of thiourea derivatives (16), the hydrogen bonding donor (HBD) at 1-N of thiourea played an important role for potency, thus, the decreasing inhibitory activities of thiazole and oxadiazole compounds can be associated with the lacking of the hydrogen bonding donor at the 1-N. Surprisingly, the HBD at 1-N containing compounds, benzothiazole (1_1) or benzimidazole (1_2) and peptidomimetics (3_1, 3_3), were also less potent than 1. It leads to a hypothesis that the structural modification causes to change the position and key interaction of these inhibitors in enzymatic active site. In particular, compound 1_2 (IC_50_ = 0.11 ± 0.03 μM) appeared to be the most potent in this group, and it exhibited 2-folds more potent than 1_1 (IC_50_ = 0.30 ± 0.09 μM), supporting the fact that the hydrogen atom of –NH in benzimidazole (1_2) might play a important role in favorable binding interaction, but further investigations are required to confirm this statement as the available data is inconclusive.

**Table 1:** IC_50_ values for inhibition of the hQC enzyme by compounds 1_x and 3_x

Cpd	Structure	IC_50_ (μM)
1_1	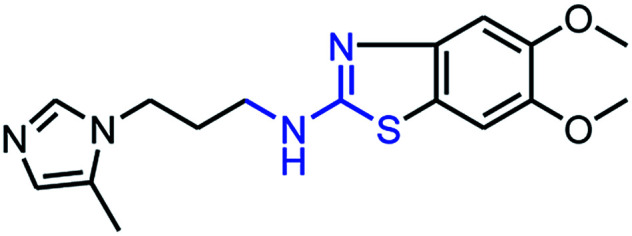	0.30 ± 0.09
1_2	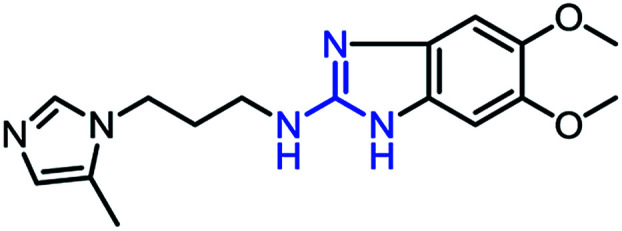	0.11 ± 0.03
1_3	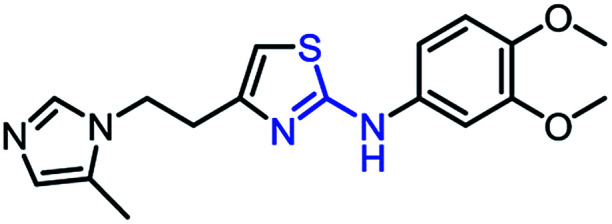	0.32 ± 0.10
1_4	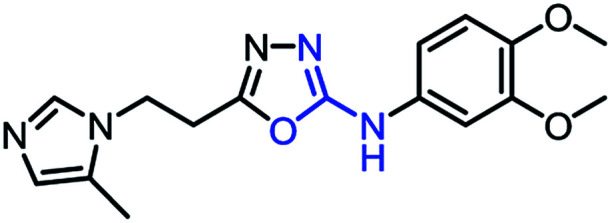	0.42 ± 0.06
3_1	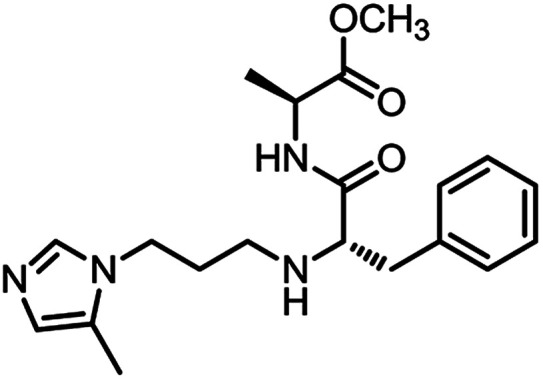	0.64 ± 0.09
3_3	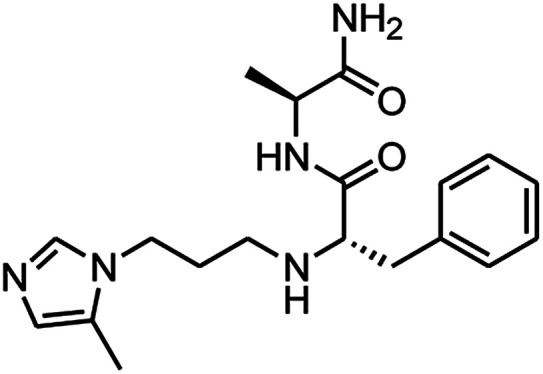	0.45 ± 0.04
1^17^	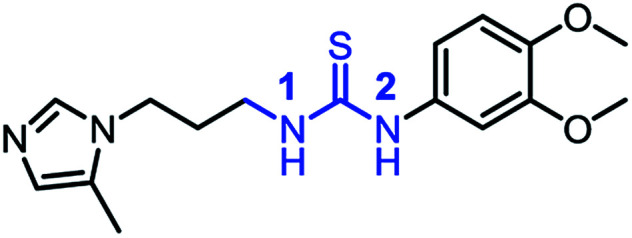	0.029 ± 0.004

### Molecular docking

**Fig. 3:**
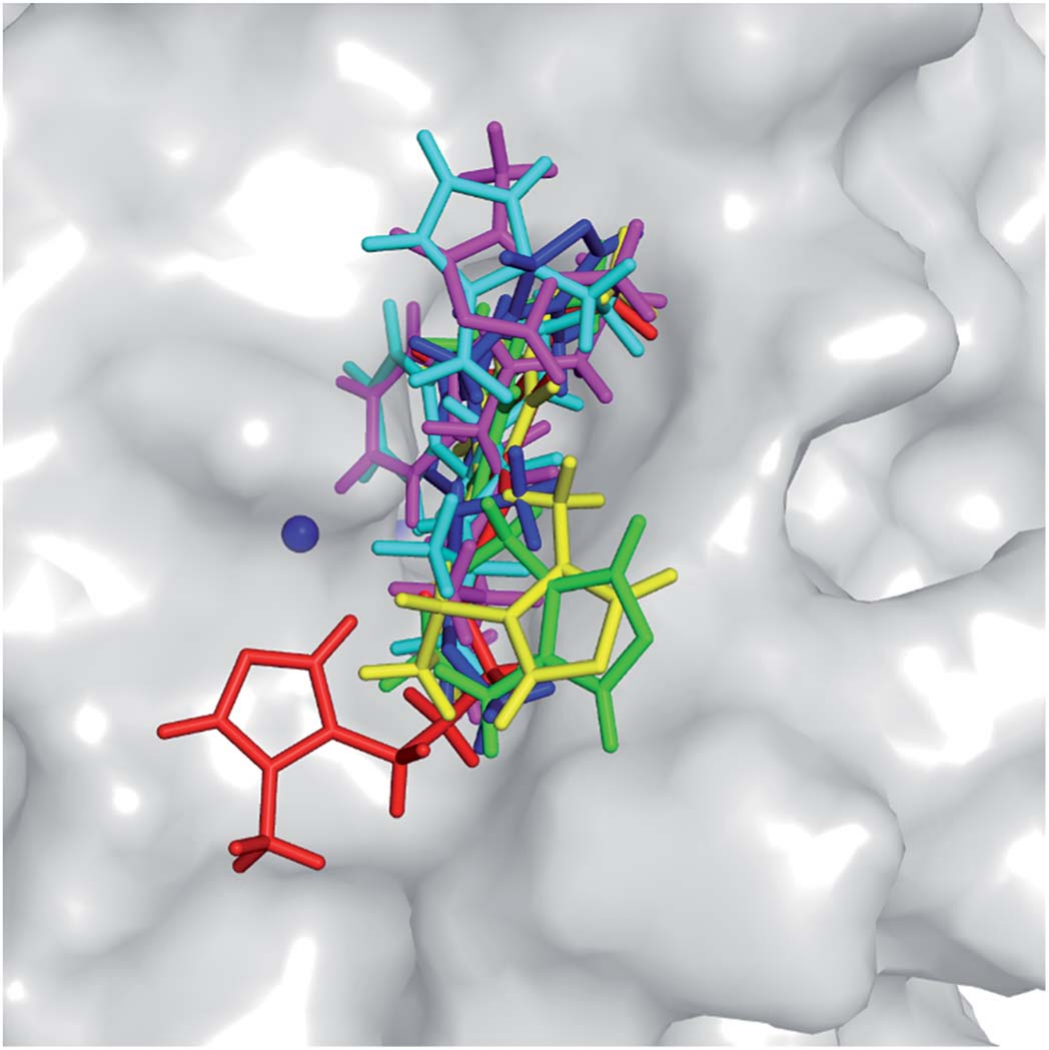
Docked conformations of potential inhibitors to the active site of the hQC enzyme (PDB ID: 3PBB) were obtained from molecular docking simulations employing Autodock4.2 package.^33^ The Zn^2+^ ion was mentioned as blue sphere.

The molecular docking approach is often employed to rapidly and roughly estimate the binding pose and binding affinity of a ligand to a protein.^52^ Autodock Vina^53^ and Autodock4 (ref. 33) are popularly employed to solve these problems. Although Autodock Vina was indicated as providing a higher accuracy with lower CPU consumption,^53^ Autodock4 showed a higher performance in this study (described in ESI file†). Autodock4 was thus employed to determine the three-dimensional structure of the hQC-inhibitor complexes. The obtained binding poses of ligands with the hQC protein are described in Fig. 3. All of the ligands completely bound to the experimental active site of the hQC enzyme. Normally, the docked complex adopts an appropriate conformation that can be used as an initial structure for atomistic MD simulations. Furthermore, the docking energies are shown in Table 2. Although the correlation between the predicted and experimental affinities was not very high since the molecular docking method did not consider the dynamics of the complexes, the coefficient was acceptable, with the value of *R*_dock_ = 0.54. The obtained results were thus refined using all-atom MD simulations.

**Table 2:** Binding affinity of potential inhibitors. Δ*E*_dock_ was provided from Autodock Vina. The absolute binding free energy Δ*G*_FEP_ was obtained by the FEP method. Δ*G*_EXP_ was approximately determined when we assumed that IC_50_ was equal to the inhibition constant (*k*_i_). The unit is of kcal mol^−1^

System	Δ*E*_dock_	Δ*G*^cou^_FEP_	Δ*G*^vdW^_FEP_	Δ*G*_FEP_	Δ*G*_EXP_
1_1	−5.1	−0.08	−9.64	−9.72 ± 0.41	−8.94
1_2	−5.7	−5.78	−6.91	−12.68 ± 0.45	−9.57
1_3	−5.7	1.92	−9.20	−7.28 ± 0.47	−8.92
1_4	−5.5	2.71	−6.72	−4.01 ± 0.40	−8.75
3_1	−4.9	0.22	−3.88	−3.66 ± 0.41	−8.50
3_3	−5.6	−2.76	−3.73	−6.49 ± 0.46	−8.68

### Refinement of docking results using MD simulations

**Fig. 4:**
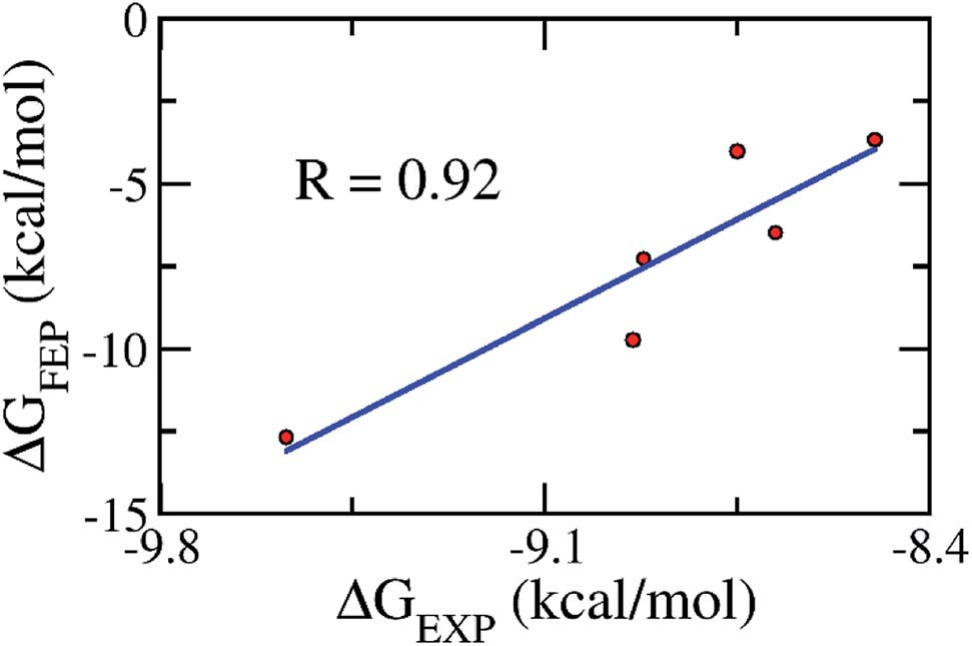
The correlation between the experimental and computational binding free energies.

As mentioned above, the molecular docking approach contains some limitations. The docked conformation was used as the initial shape for the all-atom MD simulation. The MD simulation was performed according to the description in the Materials & methods section. Six complexes were independently mimicked three times through MD simulations. The time dependence of the RMSD of the six complexes is described in Fig. S3 of the ESI file.† The systems mostly reached equilibrium states after approximately 20 ns. The coordination links between Zn(ii) and its ligands are also stable after ∼20 ns (Fig. S4†). The last snapshots of MD simulations were used as the initial conformations for FEP simulations.^54^ Moreover, six systems involving isolated ligands in solution were also simulated over three independent MD simulations of 2 ns in length to provide stable snapshots of the solvated ligand system for FEP calculations. The demolishing free energy of the ligands from two systems including the solvated complex and ligand was estimated using the BAR method.^46^ The different free energy is the binding free energy of the ligand to the enzyme, shown in Table 2. A high correlation coefficient between the experiments and FEP simulations was obtained, with the value of *R* = 0.92 (Fig. 4). On average, the theoretical binding free energy (〈Δ*G*_FEP_〉 ≈ −7.30 kcal mol^−1^) was slightly underestimated compared to that in the experimental study (〈Δ*G*_EXP_〉 ≈ −8.89 kcal mol^−1^). Here, it is noted that the IC_50_ was assumed as equal to the inhibition constant *k*_i_. This assumption probably caused the difference between the calculated and experimental values. Additionally, the difference may come from the inaccuracy of simulating the interaction energy between the component molecules including protein, ligand and solution molecules.^55,56^ Furthermore, the vdW interaction free energy Δ*G*^vdW^_FEP_ (≈−6.68 kcal mol^−1^ on average) dominates over the Coulomb interaction free energy Δ*G*^cou^_FEP_ (≈−0.63 kcal mol^−1^) during the binding process of the ligand to the hQC enzyme. A compound forming a larger vdW interaction with the hQC target probably has a stronger binding affinity.

**Fig. 5:**
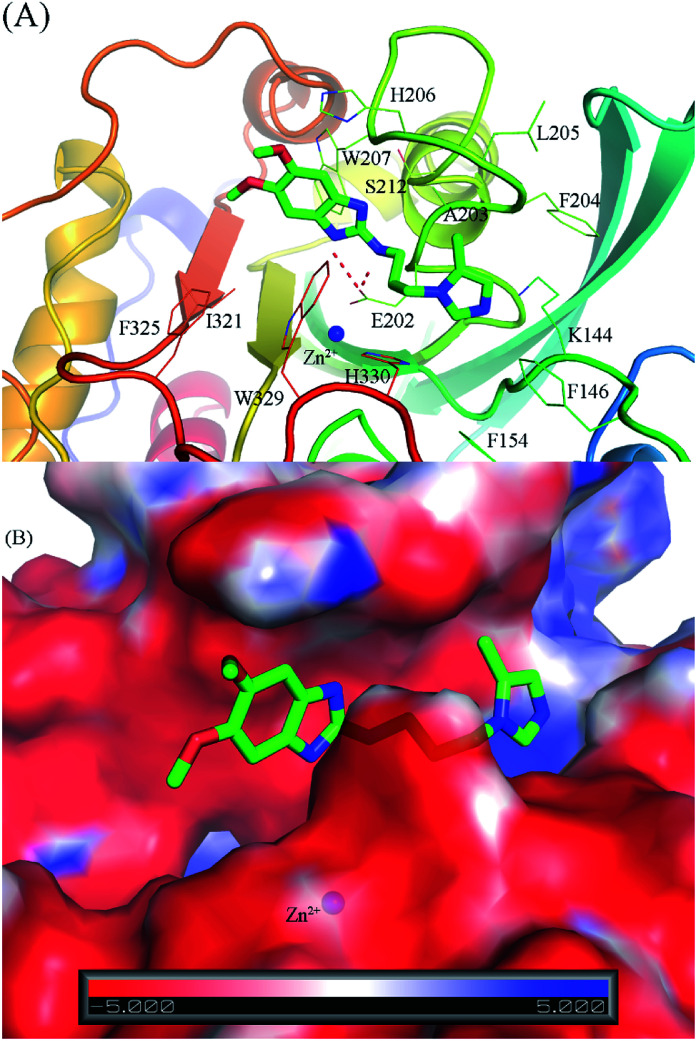
The binding pose of compound 1_2 with the hQC enzyme obtained over 50 ns of MD simulations. (A) The critical residues of hQC form sidechain and hydrogen bond contacts with compound 1_2; (B) the charged surface of the hQC enzyme in the binding mode with compound 1_2.

Through the combination of theoretical and experimental results, compound 1_2 was indicated as the most potent inhibitor of the hQC enzyme. In particular, although other compounds formed strong vdW-interaction free energy and very weak electrostatic interaction energy with the hQC enzyme, the best inhibitor 1_2 adopted a balance of electrostatic (Δ*G*^cou^_FEP_ = −5.78 kcal mol^−1^) and vdW interaction free energies (Δ*G*^vdW^_FEP_ = −6.91 kcal mol^−1^), as described in Table 2. In detail, compound 1_2 formed two HBs to residue E202 of the hQC protein by two hydrogen atoms of NH in thiourea motif (Fig. 5A). The new interaction of 2-N with enzyme caused its conformational change and can explain the similar activity of 1_1, 1_3 and 1_4, and weaker than 1_2 on hQC. Consequently, 14 residues were found that they adopt SC contacts with the ligand (Fig. 5A). Moreover, the charged surface of the hQC enzyme was analyzed using the APBS package, and the obtained result is shown in Fig. 5B. The active site of the hQC enzyme mostly adopted a negatively charged surface. The compound 1_2 fits well into the active site, although the net charge of the compound is zero. This may explain why the replacement of the S atom in compound 1_1 could significantly increase the binding affinity of compound 1_2 (Table S3†). Moreover, the quantum chemical calculations were performed to roughly evaluate the effect of ion Zn^2+^ on the binding affinity of compound 1_2 to the hQC enzyme. The details of computations were mentioned in the ESI file.† The computational modeling was described in Fig. S2 and S3 of the ESI file.† The different energy between two cases, when Zn^2+^ is appearance and disappearance, is approximate of 4.19 kcal mol^−1^, occupying ∼13% of total interaction energy. Obtained results indicate that the strong influence of Zn^2+^ on ligand-affinity. However, the Zn^2+^ adopts repulsive force to the ligand 1_2 indicating that the total net charge of the potential ligand should not be positive. Overall, designing a new inhibitor for the hQC enzyme should be carefully considered to address this problem.

An AD drug candidate is critically required to permeate the blood–brain barrier (BBB) to transfer from the circulatory system into the nervous system.^57^ Five physicochemical properties of six compounds, including MW, log *P*, log *D*, p*K*_a_, pTSA, and multiparameter optimization (mPO) were calculated and compared with compound 1 and suggested CNS drugs (Table 3).^58^ Six compounds have all molecular weight below 450 dalton, log *P* below 5, log *D* in 1-4 range and p*K*_a_ below 10. Compounds 1_1 to 1_4 are also in CNS drugs range of pTSA, meanwhile pTSA of 3_1 and 3_3 are slightly larger than the suggestion value. p*K*_a_ value of all 1_x series compounds are below 8.0, indicating that they avoid possible interactions with P-gp.^58^ For mPO score, six compounds pass the recommendation score for CNS drug (mPO score > 4). In general, 1_x series and reference compound 1 are in of the rule of CNS drugs, confirmed their estimations in BBB penetration below. However, PDB150, a 1 derivative, was demonstrated to impossible permeation the BBB,^28^ indicating the same result of 1 on BBB penetration study. Therefore, log(BBB) was employed for more BBB information. Moreover, the PreADME package^50^ was employed to predict the BBB-permeating ability, termed log(BBB). A compound is able to cross the BBB when the log(BBB) is within the range from −2 to 1.^59^ The obtained results of the log(BBB) for the six compounds are mentioned in Table 3. The predicted log(BBB) values ranged from −1.44 to −0.24, indicating that all of these compounds are able to cross the BBB. Inhibitor 1_1 is the compound best capable of penetrating the BBB with the log(BBB) of −0.24. Meanwhile, inhibitor 1_2 also shows the ability to penetrate the BBB impressively, with the log(BBB) of −0.44. And, the low log(BBB) of 1 (−1.38) can explain for low predicted BBB penetration. Moreover, the human intestinal absorption (HIA) capability was also evaluated to predict the percentage of a drug candidate absorbed through the gastrointestinal tract. The obtained results indicated that all six inhibitors can be used as an oral drug, with HIA values mostly larger than 89% (Table 3).

**Table 3:** Predicted blood–brain barrier permeation and human intestinal absorption obtained using the PreADME application

System	MW	log *P*	log *D*	p*K*_a_	pTSA (Å^2^)	mPO	log(BBB)	HIA (%)
1	344.44	2.25	2.11	7.33	60.35	5.44	−1.38	94.72
1_1	332.42	2.91	2.80	7.08	61.21	5.43	−0.24	90.74
1_2	315.38	2.12	1.98	7.86	77.00	5.50	−0.40	89.30
1_3	344.43	3.02	2.92	7.07	51.21	5.36	−1.24	97.15
1_4	329.36	2.17	2.10	7.07	87.25	5.78	−1.13	95.94
3_1	372.47	1.33	0.96	8.79	85.26	5.02	−1.44	94.27
3_3	357.46	0.43	0.56	8.49	99.48	4.44	−1.17	84.95
CNS drugs	<450	<5	1–4	<10	<90	>4	−2 to 1	

## Conclusions

*In vitro* and *in silico* studies indicated that compound 1_2 is a high-potential inhibitor of the hQC enzyme for the prevention of AD. Although almost ligands formed strong vdW and weak electrostatic interactions with the hQC enzyme, compound 1_2 adopted a balance of the two interactions. E202 is the most important residue, forming a critical hydrogen bond with the ligand 5,6-dimethoxy-*N*-(3-(5-methyl-1*H*-imidazol-1-yl)propyl)-1*H*-benzo[*d*]imidazol-2-amine (1_2), which probably controls the binding process of the complex. Furthermore, replacing a chemical element of compound 1_2 with a positively charged chemical element could improve the binding affinity the designed ligand, but the total net charge of the ligand should be zero to reduce repulsive force from ion Zn^2+^. The compound 1_2 was predicted to be able to permeate the BBB, and it can be used as an oral drug, as a high HIA was estimated. An *in vivo* study should be carried out to confirm these results.

## Conflicts of interest

There are no conflicts to declare.

## Supplementary Material

RA-009-C9RA05763C-s001
