# Supplementary material for: In vitro and in silico determination of glutaminyl cyclase inhibitors
Source: RSC Adv. 2019 Sep 19;9(51):29619–27. doi: 10.1039/c9ra05763c (PMC9071946; doi:10.1039/c9ra05763c)
Supplement: RA-009-C9RA05763C-s001 [file RA-009-C9RA05763C-s001.pdf]

Electronic Supplementary Material (ESI) for RSC Advances.
This journal is © The Royal Society of Chemistry 2019

Supporting information for

In vitro and *in silico* Determinations of Glutaminyl Cyclase Inhibitors

Phuong-Thao Tran[1¶*], Van-Hai Hoang[2¶], Jeewoo Lee[3], Tran Thi Thu Hien[4], Nguyen Thanh Tung[5], and Son Tung Ngo[6,7*]

[1]Department of Pharmaceutical Chemistry, Hanoi University of Pharmacy, Hanoi, Vietnam;
[2]Institute of Research and Development, Duy Tan University, Da Nang 550000, Vietnam;
[3]Laboratory of Medicinal Chemistry, College of Pharmacy, Seoul National University, Seoul, Korea;
[4]Vietnam University of Traditional Medicine, Hanoi, Vietnam;
[5]Institute of Materials Science, Vietnam Academy of Science and Technology, Hanoi, Vietnam;
[6]Laboratory of Theoretical and Computational Biophysics, Ton Duc Thang University, Ho Chi Minh City, Vietnam;
[7]Faculty of Applied Sciences, Ton Duc Thang University, Ho Chi Minh City, Vietnam.

[¶]These authors contributed equally to this work

Email: thaotp119@gmail.com and ngosontung@tdtu.edu.vn

Chemistry

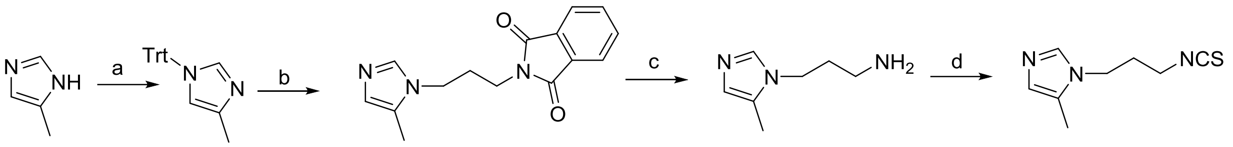

Scheme 3. 1-(3-Isothiocyanatopropyl)-5-methyl-1*H*-imidazole synthetic route. Reagents and conditions. (a) TrtCl, DMF, 0 ^{o}C to r.t., 4h; (b) *N*-(3-bromopropyl)phthalimide, MeCN, reflux, 24h then MeOH, THF, reflux, o.n. (c) $N_2H_4.H_2O$, EtOH, r.t., o.n.; (d) di-2-pyridyl-thionocarbonate, DCM, 3h, r.t (1).

5,6-Dimethoxybenzo[d]thiazol-2-amine (Ib). Acetic acid was added to a mixture of 3,4-dimethoxyaniline (1 equiv) and KSCN (1.2 equiv) in a round-bottom flask. Then, the mixture was cooled to 0- 5 °C, and a solution of Br_2 (1 equiv) in acetic acid was added dropwise. The mixture was removed from the ice bath, and the solution was stirred for 12 h. Afterward, the mixture was diluted with water and basified with 1 N NaOH, and the formed solid was collected, dried and purified by silica gel column chromatography to obtain a yellow solid. Yield: 41%. ^{1}H NMR (300 MHz, CD_3OD): δ 7.20 (s, 1H), 7.00 (s, 1H), 3.83 (s, 3H), 3.81 (s, 3H).

2-Chloro-5,6-dimethoxybenzo[d]thiazole (Ic). Isoamylnitrite (2.0 equiv) was added dropwise to cooled (water-ice bath) **Ib** (1.0 equiv) and copper (II) chloride (1.5 equiv) in acetonitrile. The mixture was stirred at 0 °C for 15 min and then at room temperature for 16 h. The reaction mixture was concentrated and then extracted with ethyl acetate and washed with brine. The organic layer was dried over $MgSO_4$ and purified by column chromatography to yield a brownish solid. Yield: 39%. ^{1}H NMR (300 MHz, $CDCl_3$): δ 7.42 (s, 1H), 7.13 (s, 1H), 3.96 (s, 3H), 3.95 (s, 3H).

5,6-Dimethoxy-N-(3-(5-methyl-1H-imidazol-1-yl)propyl)benzo[d]thiazol-2-amine (1_1). A mixture of $Pd(OAc)_2$ (0.05 %mol), sodium *tert*-butoxide (2.0 equiv), **Ic** (1 equiv) was placed in a two-neck round-bottom flask. To the flask was added dimethylacetamide and then the flask was degassed and refilled with nitrogen. Then, a solution of $P(Cy)_3$ (0.05 %mol) and 3-(5-methyl-1*H*-imidazol-1-yl)propan-1-amine (1.0 equiv) in DMA was added to the reaction mixture. The mixture was heated to 100 °C for 12 h, then cooled and diluted with water. The aqueous solution was extracted with EA 2 times, and the combined organic phase was washed water (2 times) and brine, dried over $MgSO_4$ and purified by silica gel column chromatography to obtain a brown solid. Yield: 33%. ^{1}H NMR (300 MHz, CD_3OD): δ 7.59 (s, 1H), 7.21 (s, 1H), 7.06 (s, 1H), 6.68 (s, 1H),

4.06 (t, J = 6.96 Hz, 2H), 3.85 (s, 3H), 3.82 (s, 3H), 3.39 (t, J = 6.78 Hz, 2H), 2.21 (d, J = 1.11 Hz, 3H), 2.11-2.07 (m, 2H). ESI-MS *m/z*: 333.0 $[M+H]^+$. MS (HR-FAB-MS): *m/z* $[M + H]^+$ calcd for $C_{16}H_{21}N_4O_2S$: 333.1385; found: 333,1387. Anal. Calcd. For $C_{16}H_{20}N_4O_2S$ (332.1307): C, 57.81; H, 6.06; N, 16.85. Found: C, 57.79; H, 6.08; N, 16.87.

4,5-Dimethoxybenzene-1,2-diamine (IIb). 1,2-Dimethoxy-4,5-dinitrobenzene (300 mg, 1.31 mmol) was dissolved in a 20 mL mixture of MeOH and tetrahydrofuran (4:1, v/v), and then 10% Pd/C was added. The mixture was stirred at room temperature under hydrogen gas until the starting material was consumed. The crude mixture was filtered through Celite, washed with methanol, concentrated, and then purified by column chromatography (MeOH/CH_2Cl_2 = 1/50) to afford 200 mg of 4,5-dimethoxybenzene-1,2-diamine as a dark green solid (90%). ^{1}H NMR (300 MHz, $CDCl_3$): δ 6.37 (s, 2H), 3.79 (s, 6H), 3.21 (br, 4H).

5,6-Dimethoxy-N-(3-(5-methyl-1H-imidazol-1-yl)propyl)-1H-benzo[d]imidazol-2-amine (1_2). To a solution of 1-(3-isothiocyanatopropyl)-5-methyl-1*H*-imidazole (70 mg, 0.38 mmol) in THF/DMF (2 mL, 1/1) at room temperature, 4,5-dimethoxybenzene-1,2-diamine (65 mg, 0.38 mmol) was added, and the contents were stirred at room temperature for 2 h. 1-Ethyl-3-(3-dimethylaminopropyl) carbodiimide hydrochloride (72 mg, 0.46 mmol) was then added to the reaction mixture, and the contents were stirred at 65-70 °C for 1 h. The reaction mixture was then cooled to room temperature and poured into ice-cold water (5 mL), and the solid was collected by filtration. The crude product thus obtained was purified by PLC using MeOH/CH_2Cl_2 (1/9) as the eluent to give the desired compound as a light yellow solid (20 mg, 17%). ^{1}H NMR (300 MHz, CD_3OD): δ 7.57 (s, 1H), 6.65 (s, 1H), 6.62 (s, 1H), 6.52 (s, 1H), 3.99 (t, J = 7.50 Hz, 2H), 3.79 (s, 3H), 3.72 (s, 3H), 3.59 (t, J = 6.39 Hz, 2H), 2.21 (d, J = 0.90 Hz, 3H), 2.02 (quint, J = 7.32 Hz, 2H). FAB-MS *m/z*: 316.2 $[M+H]^+$. MS (HR-FAB-MS): *m/z* $[M + H]^+$ calcd for $C_{16}H_{21}N_5O_2$: 316.1768; found: 316.1772. Anal. Calcd. For $C_{16}H_{21}N_5O_2$ (315.1695): C, 60.94; H, 6.71; N, 22.21. Found: C, 60.92; H, 6.74; N, 22.23.

Ethyl 2-(2-chlorothiazol-4-yl) acetate (IIIb). Compound **IIIb** was prepared by following the procedure of **Ic** from IIIa to yield the desired product (280 mg, 51%) as a yellow oil. ^{1}H NMR (300 MHz, $CDCl_3$): δ 7.11 (t, J = 0.72 Hz, 1H), 4.22 (q, J = 7.14 Hz, 2H), 3.76 (d, J = 0.75 Hz, 2H), 1.29 (t, J = 7.14 Hz, 3H).

Ethyl 2-(2-(3,4-dimethoxyphenylamino)thiazol-4-yl) acetate (IIIc). To a solution of ethyl 2-(2-chlorothiazol-4-yl) acetate (100 mg, 0.49 mmol) in ethanol, *p*-toluene sulfonic acid monohydrate (93 mg, 0.49 mmol) was added. The reaction mixture was refluxed for 4 h, then 3,4-dimethoxyaniline (112 mg, 0.73 mmol) was added and refluxed overnight. The solvent was removed by evaporation and then purified by column chromatography (EA/n-hexane = 1/4) to yield the desired product (57 mg, 36%) as a yellow oil. ^{1}H NMR (400 MHz, $CDCl_3$): δ 6.92 (d, J = 2.04 Hz, 1H), 6.83-6.77 (m, 2H), 6.31 (s, 1H), 4.12 (q, J = 7.16 Hz, 2H), 3.81 (s, 6H), 3.54 (s, 2H), 1.22 (t, J = 7.12 Hz, 3H).

2-(2-(3,4-Dimethoxyphenylamino)thiazol-4-yl) ethanol (IIId). A solution of ethyl 2-(2-(3,4-dimethoxyphenylamino)thiazol-4-yl) acetate (602 mg, 1.87 mmol) in anhydrous THF was dropwise added to lithium aluminum hydride (107 mg, 2.8 mmol) in anhydrous THF at 0 °C. The reaction mixture was stirred at 0 °C for 1 h. Afterward, brine was added slowly at 0 °C and then removed THF by evaporation, and the solution was extracted with ethyl acetate and water. The extracted organic layer was dried over Na_2SO_4, and the ethyl acetate was removed by evaporation. The reaction mixture was purified by column chromatography (EtOAc/n-hexane = 4/5) to obtain 320 mg of the desired product as a brown oil (61% yield). ^{1}H NMR (300 MHz, $CDCl_3$): δ 6.97 (d, J = 1.08 Hz, 1H), 6.84-6.83 (m, 2H), 6.21 (s, 1H), 3.92 (t, J = 5.70 Hz, 2H), 3.88 (d, J = 1.83 Hz, 6H), 2.83 (t, J = 5.13 Hz, 2H), 1.71-1.67 (m, OH).

4-(2-Bromoethyl)-N-(3,4-dimethoxyphenyl)thiazol-2-amine (IIIe). To a solution of 2-(2-(3,4-dimethoxyphenylamino)thiazol-4-yl) ethanol (320 mg, 1.14 mmol) in THF, tetrabromomethane (455 mg, 1.37 mmol) and triphenylphosphine (359 mg, 1.37 mmol) were added. The reaction mixture was stirred at room temperature for 15 h and then extracted with ethyl acetate and water. The organic layer was dried over $MgSO_4$ and purified by column chromatography (EA/n-hexane = 3/10) to yield the product as a light yellow solid (125 mg, 33%). ^{1}H NMR (400 MHz, $CDCl_3$): δ 7.14 (br, NH), 6.95 (s, 1H), 6.83 (s, 2H), 6.25 (s, 1H), 3.86 (d, J = 1.88 Hz, 6H), 3.66 (t, J = 7.20 Hz, 2H), 3.12 (t, J = 7.04 Hz, 2H).

N-(3,4-dimethoxyphenyl)-4-(2-(5-methyl-1H-imidazol-1-yl)ethyl)thiazol-2-amine (1_3). 4-(2-Bromoethyl)-*N*-(3,4-dimethoxyphenyl)thiazol-2-amine (117 mg, 0.34 mmol) was suspended in 10 mL acetonitrile, and 5-methyl-1-trityl-1*H*-imidazole (133 mg, 0.41 mmol) was added. The

mixture was kept under reflux overnight. The organic solvent was removed to yield the mixture. The crude mixture was dissolved in a stirred solution containing methanol (5 mL) and trifluoroacetic acid (1.98 mL). The mixture was kept under reflux overnight. The solvent was removed, and the remaining oil was purified by flash chromatography using silica gel and an MC/MeOH gradient to afford the product as a light brown solid. Yield: 45 mg (39%, 2 steps). ^1H NMR (300 MHz, CD_3OD): *δ* 7.39 (d, *J* = 1.11 Hz, 1H), 7.26 (d, *J* = 2.40 Hz, 1H), 7.01 (dd, J_1 = 8.61, 2.40 Hz, 1H), 6.91 (d, *J* = 8.61 Hz, 1H), 6.61 (s, 1H), 6.20 (s, 1H), 4.27 (t, *J* = 6.75 Hz, 2H), 3.83 (s, 3H), 3.80 (s, 3H), 2.97 (d, *J* = 6.60 Hz, 2H), 2.16 (d, *J* = 1.11 Hz, 3H). FAB-MS *m/z*: 345 $[M+H]^+$. MS (HR-FAB-MS): *m/z* $[M + H]^+$ calcd for $C_{17}H_{20}N_4O_2S$: 345.1385; found: 345.1385. Anal. Calcd. For $C_{17}H_{20}N_4O_2S$ (344.1307): C, 59.28; H, 5.85; N, 16.27. Found: C, 59.25; H, 5.87; N, 16.29.

Methyl 3-(5-methyl-1H-imidazol-1-yl)propanoate (IVb). Compound **IVb** was prepared by following the synthetic process of **1_3** from **IVa**. ^1H NMR (300 MHz, $CDCl_3$): 7.45 (s, 1H), 6.77 (s, 1H), 4.17 (t, *J* = 6.96 Hz, 2H), 3.70 (s, 3H), 2.74 (t, *J* = 6.96 Hz, 2H), 2.22 (d, *J* = 0.93 Hz, 3H).

3-(5-Methyl-1H-imidazol-1-yl)propanehydrazide (IVc). To the solution of **IVb** (1 equiv) in EtOH was added hydrazine monohydrate (5.0 equiv), and the mixture was stirred at room temperature overnight. The reaction mixture was concentrated and used for the next step without purification. ^1H NMR (300 MHz, CD_3OD): *δ* 7.50 (s, 1H), 6.65 (s, 1H), 4.22 (t, *J* = 6.60 Hz, 2H), 2.56 (t, *J* = 6.57 Hz, 2H), 2.22 (d, *J* = 0.90 Hz, 3H).

N-(3,4-Dimethoxyphenyl)-2-(3-(5-methyl-1H-imidazol-1-yl)propanoyl)hydrazine-1-carboxamide (IVd). To a solution of 3,4-dimethoxyaniline (1.0 equiv) in 5 mL DMC, 1,1'-carbonyldiimidazole was added slowly, and the mixture was stirred at room temperature for 1 h. Then, a solution of **IVc** (1.0 equiv) in 5 mL DMF was added to the reaction mixture. The reaction mixture was stirred overnight, concentrated and dissolved with EA. The organic solution was washed with water and brine, dried over $MgSO_4$, concentrated and purified by silica gel column chromatography to yield a white solid. ^1H NMR (300 MHz, DMSO): *δ* 9.76 (s, 1H), 8.54 (s, 1H), 8.02 (s, 1H), 7.51 (s, 1H), 7.13 (d, *J* = 2.40 Hz, 1H), 6.91 (dd, *J* = 8.61, 2.22 Hz, 1H), 6.84 (d, *J* = 8.61 Hz, 1H), 6.60 (s, 1H), 4.11 (t, *J* = 6.96 Hz, 2H), 3.71 (s, 3H), 3.69 (s, 3H), 2.60 (t, *J* = 6.93 Hz, 2H), 2.17 (s, 3H).

N-(3,4-dimethoxyphenyl)-5-(2-(5-methyl-1H-imidazol-1-yl)ethyl)-1,3,4-oxadiazol-2-amine (1_4). A solution of **IVd** in 2 mL PBr_3 was refluxed for 2 h under a nitrogen atmosphere. Ice was added to the reaction mixture, followed by sodium carbonate solution to obtain a basic medium (pH = 9). The aqueous solution was extracted by EA (two times). The organic phase was collected, washed with water, dried over $MgSO_4$ and concentrated. The residue was purified by silica gel column chromatography (MeOH: DCM 1/19) to obtain a white solid. ^{1}H NMR (300 MHz, DMSO): δ 10.19 (s, 1H), 7.53 (s, 1H), 7.20 (s, 1H), 7.02 (d, J = 7.32 Hz, 1H), 6.90 (d, J = 8.79 Hz, 1H), 6.59 (s, 1H), 4.24 (t, J = 6.78 Hz, 2H), 3.73 (s, 3H), 3.70 (s, 3H), 3.18 (t, J = 6.57 Hz, 2H), 2.14 (s, 3H). ESI-MS *m/z*: 330.0 $[M+H]^+$. Anal. Calcd. For $C_{16}H_{19}N_5O_3$ (329.1488): C, 58.35; H, 5.81; N, 21.26. Found: C, 58.33; H, 5.84; N, 21.28.

Methyl (tert-butoxycarbonyl)-L-phenylalanyl-L-alaninate (Vb). Alanine methyl ester hydrochloride (1 equiv), EDC.HCl (1.2 equiv), TEA (3 equiv) and HOBt (1.2 equiv) were dissolved in methylene chloride. After stirring for 5 min at room temperature, a carboxylic acid derivative (1.2 equiv) was added to the reaction solution. The reaction mixture was stirred overnight at room temperature. Then, the mixture was washed with water, dried over $MgSO_4$, concentrated and purified by column chromatography. Yield 90%. ^{1}H NMR (300 MHz, $CDCl_3$) δ 7.30-7.19 (m, 5H), 6.37 (br, 1H), 4.98 (s, 1H), 4.52 (quintet, J = 7.14 Hz, 1H), 4.34 (br, 1H), 3.71 (s, 3H), 3.07 (t, J = 6.06 Hz, 2H), 1.41 (s, 9H), 1.35 (d, J = 7.14 Hz, 3H).

Methyl L-phenylalanyl-L-alaninate (Vc). To a solution of the starting material (**Vb**) in methylene chloride (MC) was added triisopropylsilane (3 drops) and TFA (10 equiv), and the mixture was stirred at room temperature overnight. The mixture was diluted with MC, basified by solution of 1 M NaOH and washed one time with water. The organic layer was dried over $MgSO_4$, concentrated and purified by column chromatography. Yield 99%. ^{1}H NMR (300 MHz, $CDCl_3$) δ 7.72 (br, 1H), 7.35-7.21 (m, 5H), 4.60 (quintet, J = 7.32 Hz, 1H), 3.75 (s, 3H), 3.65 (dd, J = 9.33, 4.02 Hz, 1H), 3.25 (dd, J = 13.74, 4.02 Hz, 1H), 2.74 (dd, J = 13.71, 9.33 Hz, 1H), 1.39 (d, J = 7.35 Hz, 3H).

Methyl ((2-nitrophenyl)sulfonyl)-L-phenylalanyl-L-alaninate (Vd). 2-Nitro benzene sulfonyl chloride (1.2 equiv) was added slowly to a solution of amine (1 equiv) in MC under ice cooling. Then, TEA (2 equiv) was added dropwise to the mixture reaction. The mixture was stirred

for 4 h at room temperature. Then, the mixture was washed with water, dried over $MgSO_4$, concentrated and purified by column chromatography. Yield 70%. ^{1}H NMR (300 MHz, $CDCl_3$) δ 7.99-7.96 (m, 1H), 7.82-7.78 (m, 1H), 7.71-7.66 (m, 2H), 7.09-7.00 (m, 5H), 6.87 (d, J = 7.14 Hz, 1H), 5.97 (d, J = 6.24 Hz, 1H), 4.51 (quintet, J = 7.32 Hz, 1H), 4.17-4.11 (m, 1H), 3.74 (s, 3H), 3.18 (dd, J = 13.92, 5.31 Hz, 1H), 2.95 (dd, J = 14.10, 8.61 Hz, 1H), 1.34 (d, J = 7.14 Hz, 3H).

Methyl N-(3-bromopropyl)-N-((2-nitrophenyl)sulfonyl)-L-phenylalanyl-L-alaninate (Ve). To a solution of sulfonamide (1 equiv) in DMF was added excess Cs_2CO_3. The mixture was stirred for 10 min at room temperature, then dibromo propane (3 equiv) was added, and the mixture was stirred at 60 °C for 6 h. Water was added to the mixture, and the mixture was extracted by ethyl acetate three times. The organic layer was collected, washed with water and brine, and then dried over $MgSO_4$, concentrated and purified by column chromatography. Yield 72%. ^{1}H NMR (300 MHz, $CDCl_3$) δ 7.90-7.87 (m, 1H), 7.71-7.56 (m, 3H), 7.10 (br, 5H), 6.91 (d, J = 6.78 Hz, 1H), 4.59 (dd, J = 8.07, 6.78 Hz, 1H), 4.56 (quintet, J = 6.96 Hz, 1H), 3.82-3.56 (m, 2H), 3.69 (s, 3H), 3.40 (t, J = 6.42 Hz, 2H), 3.34 (dd, J = 13.92, 8.25 Hz, 1H), 2.93 (dd, J = 13.92, 6.78 Hz, 1H), 2.28-2.17 (m, 2H), 1.36 (d, J = 7.14 Hz, 3H).

Methyl N-(3-(5-methyl-1H-imidazol-1-yl)propyl)-N-((2-nitrophenyl)sulfonyl)-L-phenylalanyl-L-alaninate (Vf). The compound was prepared from compound **Ve** by following a similar procedure to that used to synthesize compound **1_3** to afford a white solid. Yield 52%. ^{1}H NMR (300 MHz, $CDCl_3$) δ 7.75 (dd, J = 7.89, 1.65 Hz, 1H), 7.67 (dd, J = 8.25, 1.47 Hz, 1H), 7.62-7.56 (m, 2H), 7.40 (d, J = 0.93 Hz, 1H), 713-7.03 (m, 6H), 6.80 (s, 1H), 4.56 (dd, J = 8.43, 6.42 Hz, 1H), 4.42 (quintet, J = 7.14 Hz, 1H), 3.87 (t, J = 7.14 Hz, 2H), 3.68 (s, 3H), 3.66-3.54 (m, 1H), 3.49-3.38 (m, 1H), 3.25 (dd, J = 13.74, 8.61 Hz, 1H), 2.84 (dd, J = 13.71, 6.57 Hz, 1H), 2.22 (d, J = 0.9 Hz, 3H), 2.17-2.04 (m, 2H), 1.32 (d, J = 7.14 Hz, 3H).

Methyl (3-(5-methyl-1H-imidazol-1-yl)propyl)-L-phenylalanyl-L-alaninate (3_1). To a mixture of starting material (1.0 equiv) and K_2CO_3 (3.0 equiv) in acetonitrile was added thiophenol (2.0 equiv), and the mixture was stirred overnight at room temperature. The mixture was diluted with water and extracted by MC. The organic phase was washed with water, dried over $MgSO_4$, concentrated and purified by column chromatography. Yield 50%. ^{1}H NMR (300 MHz, $CDCl_3$) δ 7.64 (d, J = 8.04 Hz, 1H), 7.36-7.20 (m, 6H), 6.74 (s, 1H), 4.67-4.57 (m, 1H), 3.88-

3.70 (m, 5H), 3.29 (dd, J = 9.51, 4.02 Hz, 1H), 3.19 (dd, *J* = 13.71, 4.02 Hz, 1H), 2.71 (dd, *J* = 13.74, 9.54 Hz, 1H), 2.60-2.45 (m, 2H), 2.13 (d, *J* = 0.9 Hz, 3H) 1.76 (quintet, *J* = 6.96 Hz, 2H), 1.40 (d, *J* = 7.14 Hz, 3H). ESI-MS *m/z*: 373.1 $[M+H]^+$. MS (HR-FAB-MS): *m/z* $[M + H]^+$ calcd for $C_{20}H_{29}N_4O_3$: 373.2240; found: 373.2237. Anal. Calcd. For $C_{20}H_{28}N_4O_3$ (372.2161): C, 64.49; H, 7.58; N, 15.04. Found: C, 64.47; H, 7.60; N, 15.07.

(S)-N-((S)-1-amino-1-oxopropan-2-yl)-2-((3-(5-methyl-1H-imidazol-1-yl)propyl)amino)-3-phenylpropanamide (3_3). The ester compound was dissolved in a solution of NH_3 in MeOH and stirred overnight. The mixture was concentrated and dried under vacuum. Yield 63%. ^{1}H NMR (300 MHz, $CDCl_3$) δ 7.59 (d, *J* = 8.79 Hz, 1H), 7.36-7.19 (m, 6H), 6.73 (s, 1H), 6.01 (br, 1H), 5.39 (br, 1H), 4.49 (quintet, *J* = 7.50 Hz, 1H), 3.81-3.75 (m, 2H), 3.28 (dd, *J* = 9.15, 4.38 Hz, 1H), 3.15 (dd, *J* = 13.53, 4.38 Hz, 1H), 2.74 (dd, *J* = 13.56, 9.15 Hz, 1H), 2.46 (t, *J* = 6.75 Hz, 2H), 2.13 (s, 3H), 1.75 (quintet, *J* = 6.78 Hz, 2H), 1.36 (d, *J* = 6.96 Hz, 3H). ESI-MS *m/z*: 358.1 $[M+H]^+$. MS (HR-FAB-MS): *m/z* $[M + H]^+$ calcd for $C_{19}H_{27}N_5O_2$: 358.2243; found: 358.2239. Anal. Calcd. For $C_{19}H_{27}N_5O_2$ (357.2165): C, 63.84; H, 7.61; N, 19.59. Found: C, 63.82; H, 7.63; N, 19.60.

Computational scheme

Autodock Vina (2) and Autodock4 (3) were popularly employed to evaluate the binding affinity and binding pose of a ligand to a protein. Although Autodock Vina was indicated that it would provide higher accuracy with lower CPU consumption (2), Autodock4 is somewhat much accurate than Vina package upon the protein target (*Ngo et al* unpublished work). Combination of six ligands with twelve available inhibitors, which were gotten from bindingdb.org database, we have benchmarked the accuracy of both Autodock Vina and Autodock4 in order to estimate the binding affinity of ligand to hQC protein. Details of Autodock4 parameter was mentioned in the Materials & Methods section. In addition, the docking parameter of Autodock Vina was referred previous study (4). In particular, the center of docking grid was selected as the center of hQC active site with the size of $20 \times 20 \times 20$ nm. The exhaustiveness was chosen as 400 referring to the previous study (5). Obtained results were described in Table S1. Interestingly, although the different docking energy providing by Autodock Vina in comparison with experiment ($\Delta\Delta E_{Adt4} =$ 1.97 kcal/mol) was smaller than that of Autodock4 ($\Delta\Delta E_{vina} = 2.21$ kcal/mol), Autodock4 adopts

a larger correlation coefficient ($R_{Adt4} = 0.64$) than that of Autodock Vina ($R_{vina} = 0.35$). Absolutely, Autodock Vina provides inaccurate results in this case since adopting a correlation coefficient is smaller than 0.5. Autodock4 provides appropriate results that could be used as initial conformation of MD simulations.

The FEP simulations were also performed to investigate the binding affinity of some ligands **2_x**, which were reported that adopt poor affinity to the hQC enzyme in experiments. The obtained absolute binding free energy confirmed that these compounds are not able to use as hQC inhibitors (Table S3).

In addition, the quantum chemical calculations were performed to estimate the influence of Zn^{2+} on the ligand-affinity. The calculation was carried out using DFT calculation with B3LYP functional at 6-31G(d,p) level of the basic set. The active site of the complex hQC-**1_2** was selected to calculate. The modeling of calculations was described in Figure S5 and S6.

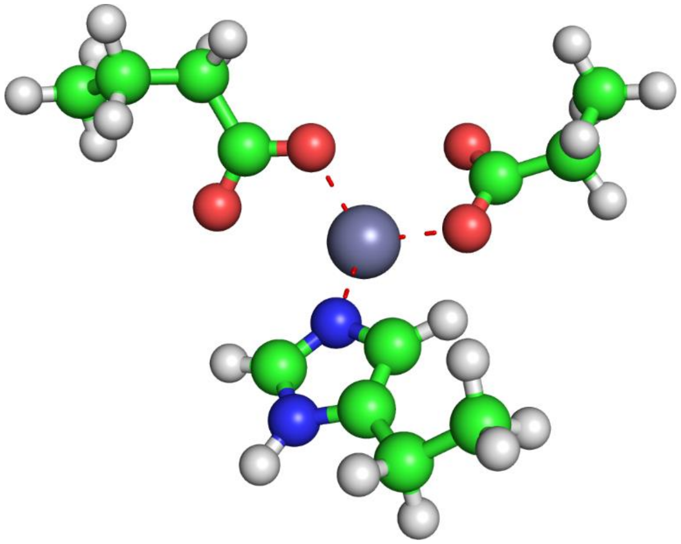

Fig. S1 The input of quantum chemical calculation at B3LYP/6-31G(d) in gas phase to estimate detail of coordination link and force constant between Zn(II) and its ligands.

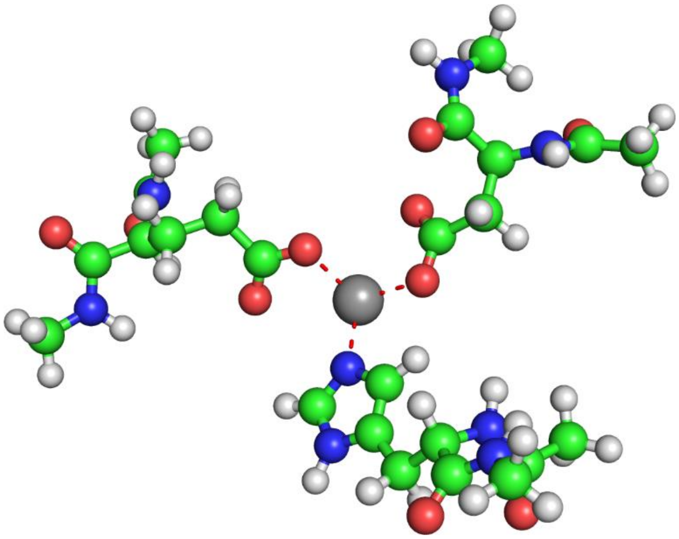

Fig. S2 The input of quantum chemical calculation at B3LYP/6-31G(d) in gas phase to estimate atomic charges of the hQC active site including Zn(II) via RESP approach.

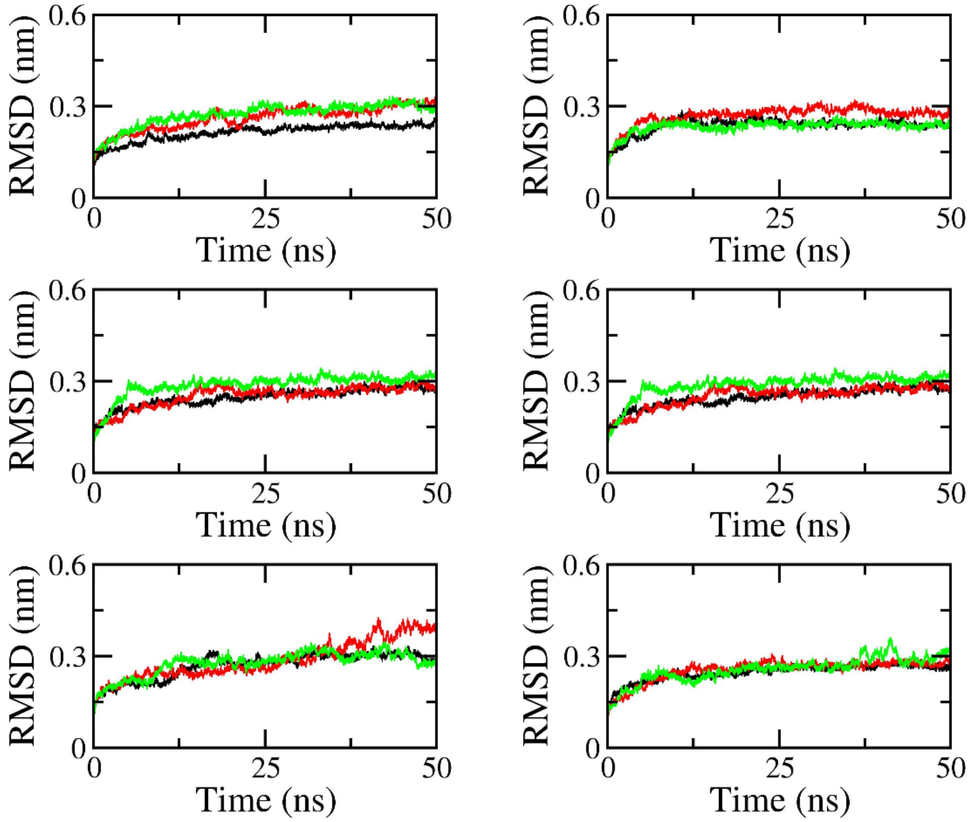

Fig. S3 The time dependence of RMSD of soluble complex over mimic time. Systems reach stable states after 20 ns, approximately.

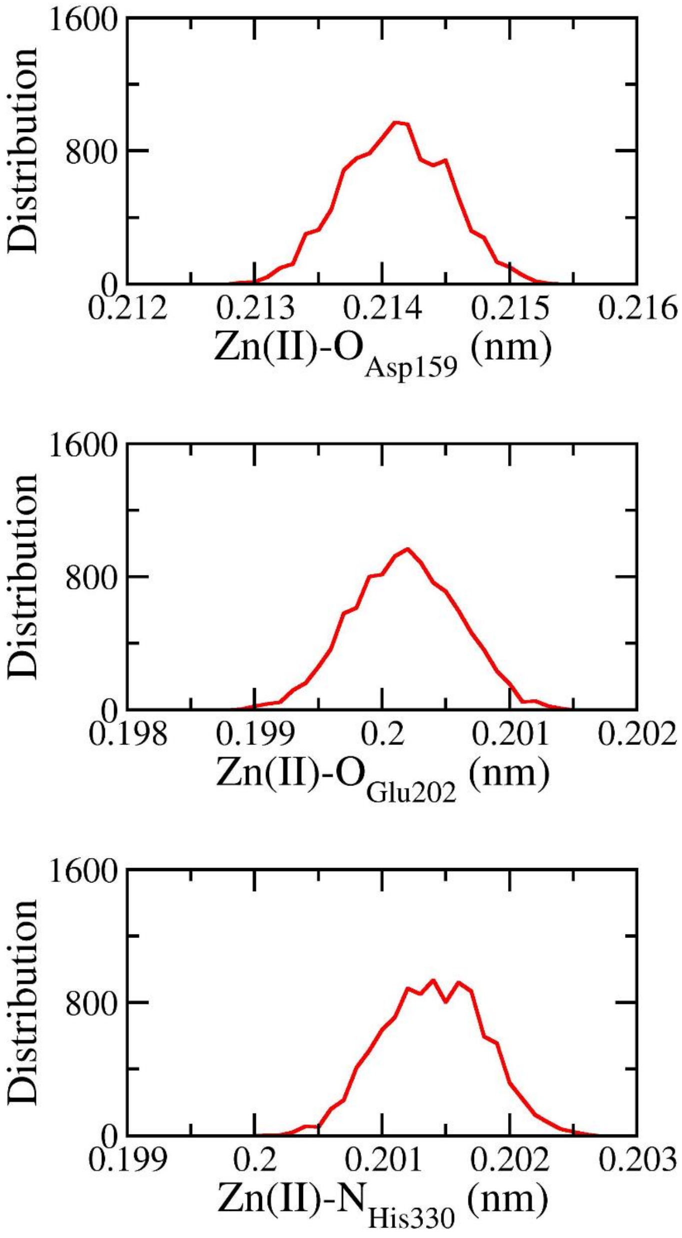

Fig. S4 The distribution of coordination links between Zn(II) and its ligands during MD simulation.

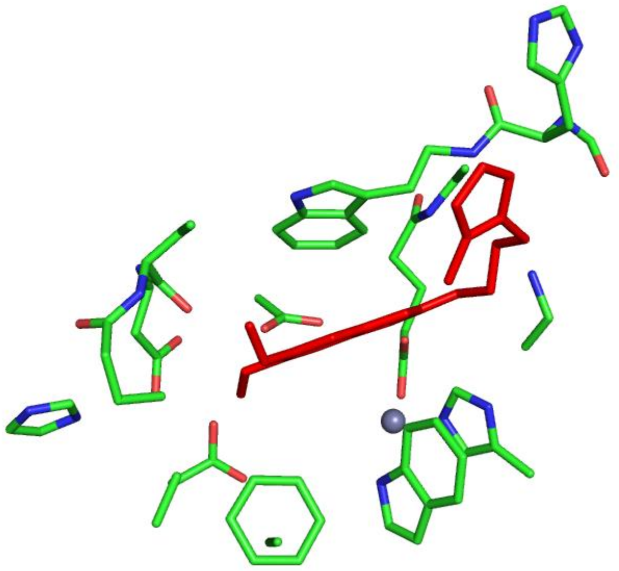

Fig. S5 The modeling of the quantum chemical calculation the interaction energy between compound **1_2** and the hQC active site including Zn^{2+}. The calculation was carried out using DFT calculations with the B3LYP functional at the 6-31G(d,p) level of basis set. The basis set superposition error was considered during the computation. Hydrogen atoms were hidden to clarify view.

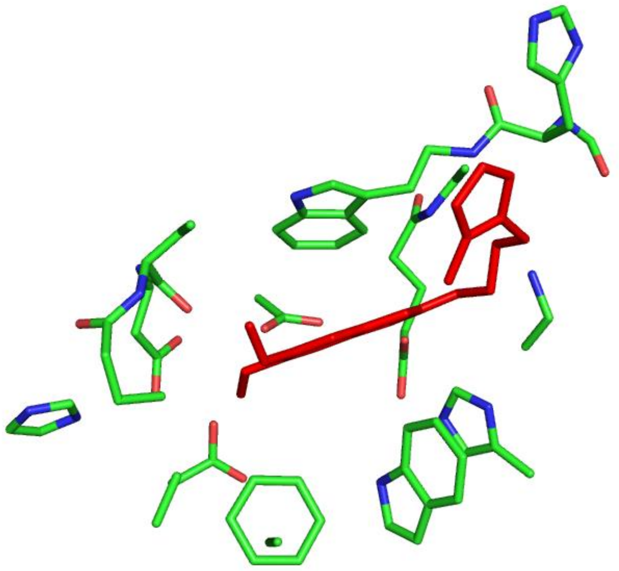

Fig. S6 The modeling of the quantum chemical calculation the interaction energy between compound **1_2** and the hQC active site without Zn^{2+}. The calculation was carried out using DFT calculations with the B3LYP functional at the 6-31G(d,p) level of basis set. The basis set superposition error was considered during the computation. Hydrogen atoms were hidden to clarify view.

Table S1. Comparison of molecular docking results of available inhibitors on hQC using Autodock Vina and Autodock4.2. The experimental values of ligands (1-12) were gotten from bindingdb.org (detail in Table S2), which compound name was chosen as the Pubchem ID. The unit is of kcal/mol.

N^0	Compound's name	ΔE_{dock}^{vina}	$\Delta E_{dock}^{Autodock4.2}$	ΔG_{EXP}
1	77918	-6.3	-5.8	-9.03
2	25062649	-6.3	-7.1	-9.91
3	44549233	-6.6	-7.7	-11.78
4	45483761	-7.1	-8	-8.95
5	45483826	-8.1	-9.5	-10.57
6	51029941	-8.6	-8.8	-10.26
7	53378967	-8.8	-7.8	-9.57
8	53379085	-8.8	-8.7	-9.16
9	53496068	-8.8	-9.4	-8.87
10	58229215	-9.1	-9.5	-10.14
11	60202587	-9.1	-9.2	-10.88
12	60202635	-9.2	-9.8	-11.47
13	1_1	-6.4	-5.1	-8.88
14	1_2	-6.5	-5.7	-9.5
15	1_3	-6.8	-5.7	-8.86
16	1_4	-7.1	-5.5	-8.69
17	3_1	-7.1	-4.9	-8.45
18	3_3	-7.4	-5.6	-8.59

Table S2. The force constant between Zn(II) and its ligands. Obtained results were obtained using quantum chemical calculation with B3LYP functional at 6-31G(d) level of basis set in gas phase.

N^0	Coordination link	Link length (Å)	Force constant (kcal/mol/Å^2)
1	Zn(II)-O_{Asp159}	2.141	50.9
2	Zn(II)-O_{Glu202}	2.000	75.0
3	Zn(II)-N_{His330}	2.014	78.0

Table S3. Detail of available compounds downloading from bindingdb.org.

N^{0}	Compounds	Ki (nM)	CID/Name
1	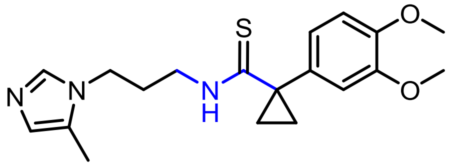	2.6	44549233
2	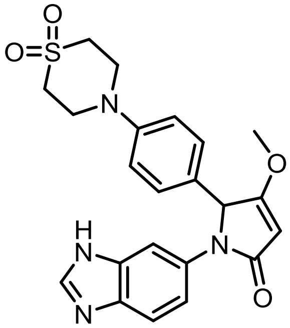	4.4	60202635
3	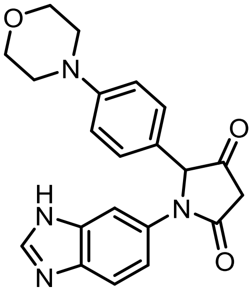	11.9	60202587
4	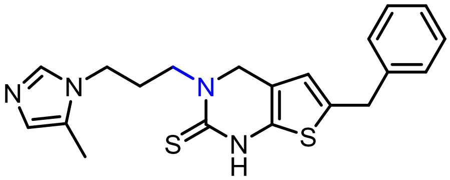	20	45483826
5	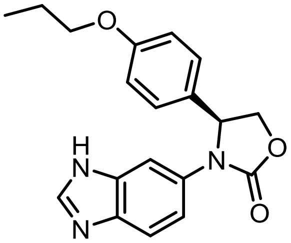	33.6	51029941
6	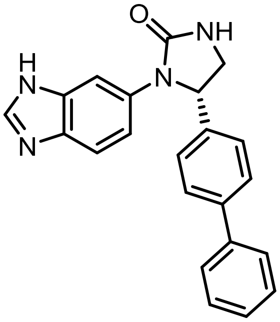	40.8	58229215

7	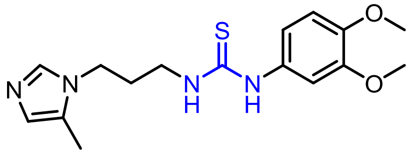	60	25062649
8	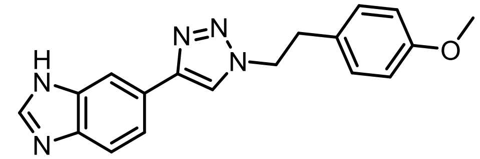	106	53378967
9	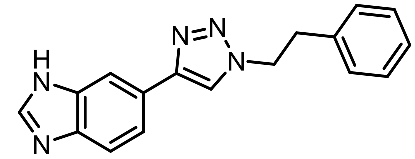	211	53379085
10	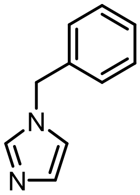	262	77918
11	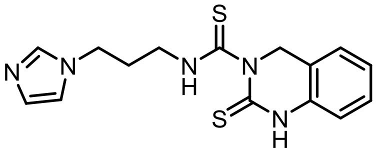	300	45483761
12	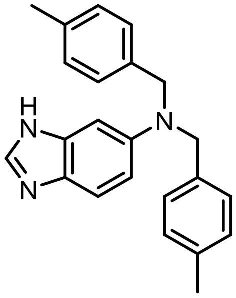	346	53496068
		**IC_{50} (nM)**	
**13**	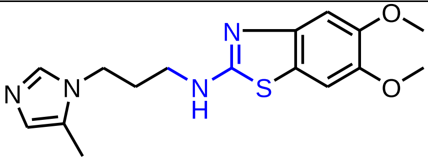	304.9 (±93.5)	**1_1**
**14**	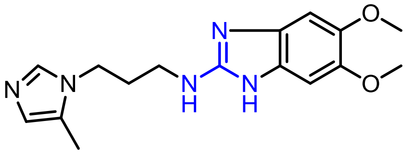	106.9 (±25.8)	**1_2**
**15**	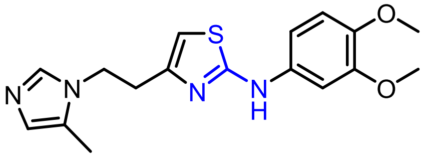	315.8 (±102.7)	**1_3**

**16**	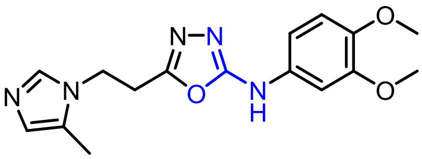	420.1 (±58.2)	**1_4**
**17**	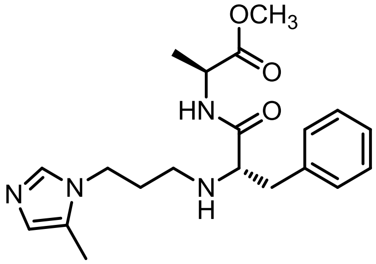	636.9 (±88.8)	**3_1**
**18**	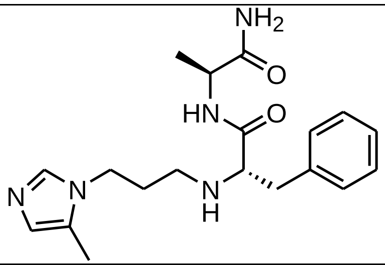	445.9 (±35)	**3_3**

Table S4. The binding free energy of inhibitor to hQC enzyme. Obtained results were provided by free energy perturbation calculation. The unit is of kcal/mol.

N^0	Compound's name	ΔG_{FEP}^{cou}	ΔG_{FEP}^{vdW}	ΔG_{FEP}	$IC50$
1	2_1	0.47	-3.26	-2.78 ± 0.78	NE
2	2_2	0.92	-2.92	-2.00 ± 0.63	NE
3	2_3	-0.68	-0.58	-1.27 ± 1.07	NE
4	2_4	1.38	-3.16	-1.78 ± 0.45	NE
5	2_5	2.07	-4.88	-2.81 ± 0.68	NE
6	2_6	4.35	-5.77	-1.42 ± 0.73	NE
7	2_7	0.26	-3.94	-3.68 ± 0.62	NE
8	2_8	2.81	-5.38	-2.57 ± 0.64	NE
9	2_9	0.91	-3.15	-2.24 ± 0.61	NE

ALL ^{1}H NMR & MS SPECTRA OF THE COMPOUNDS

^{1}H NMR of compound 1_1

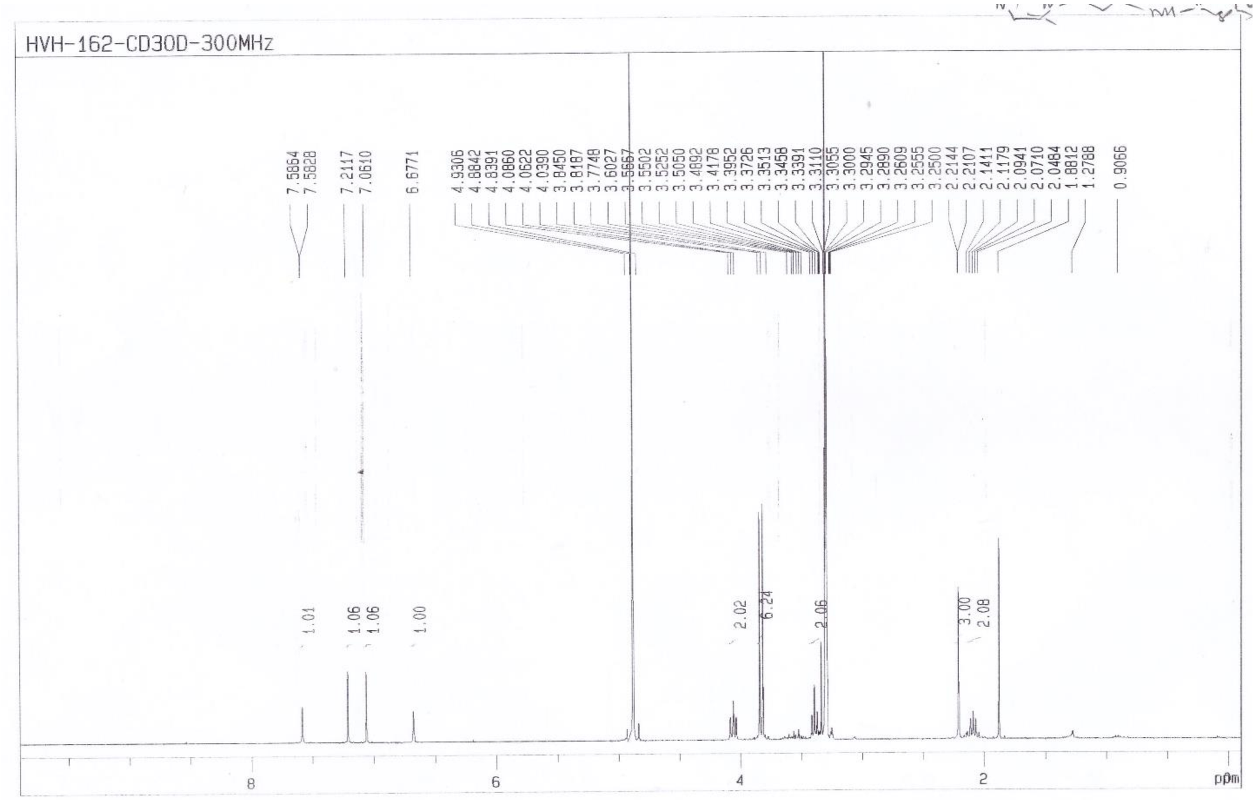

MS of compound 1_1

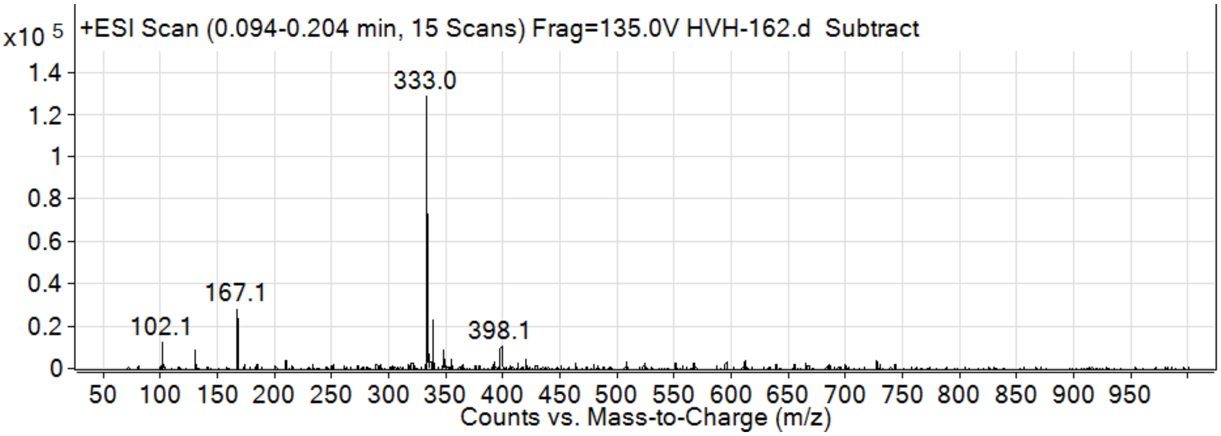

^{1}H NMR of compound 1_2

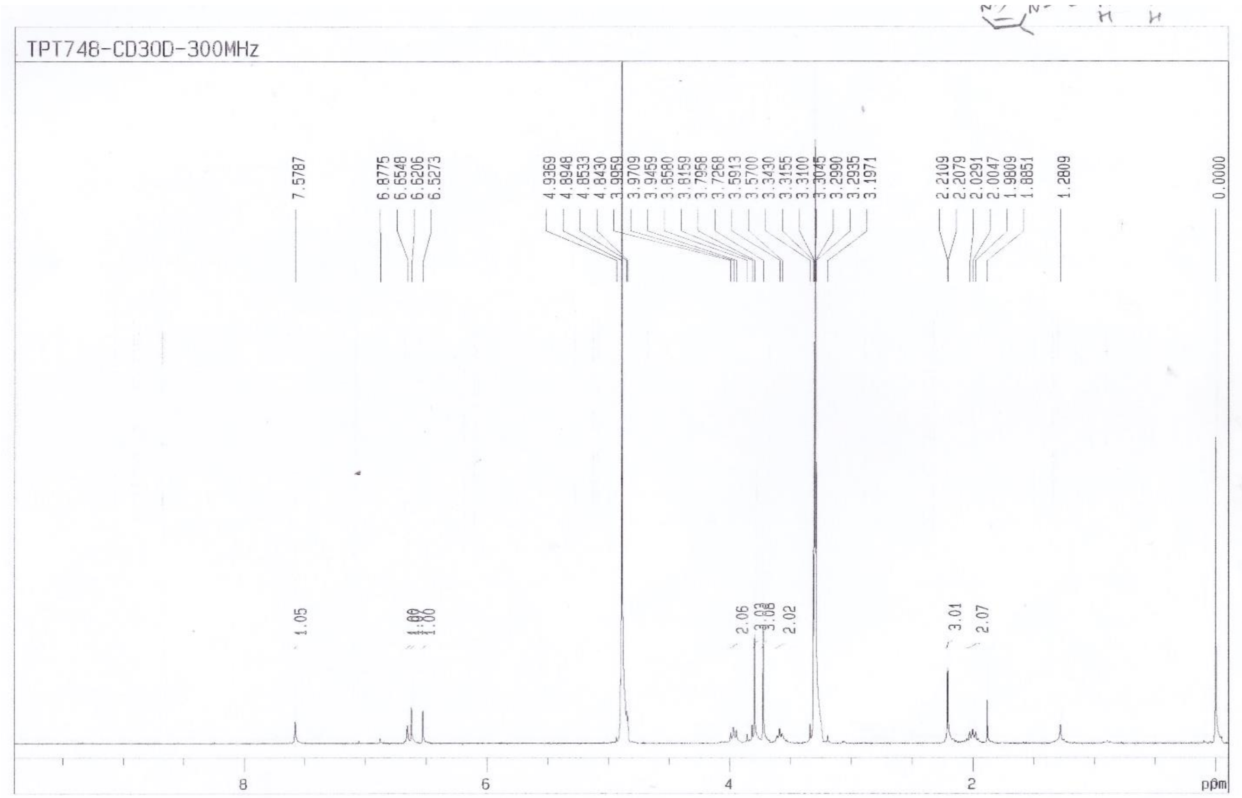

MS of compound 1_2

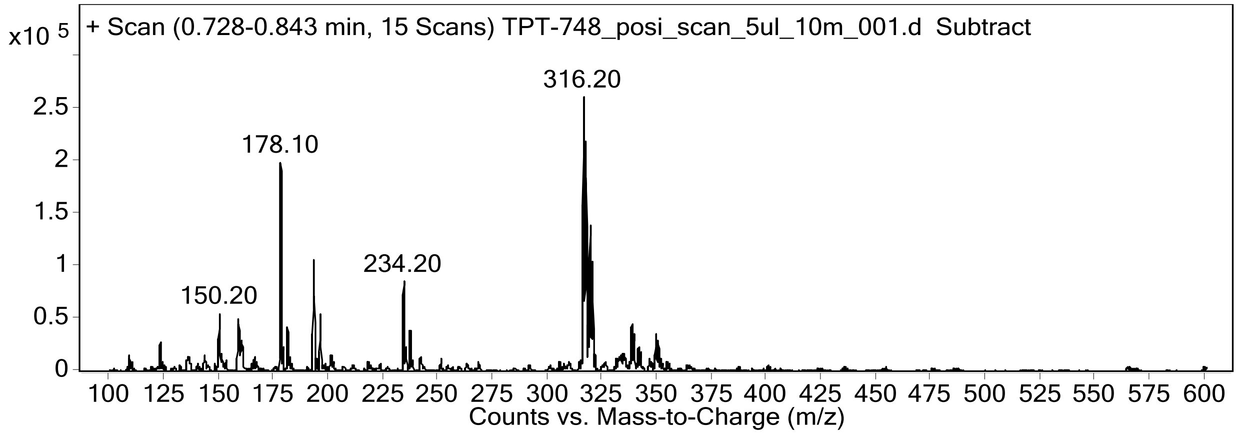

^{1}H NMR of compound 1_3

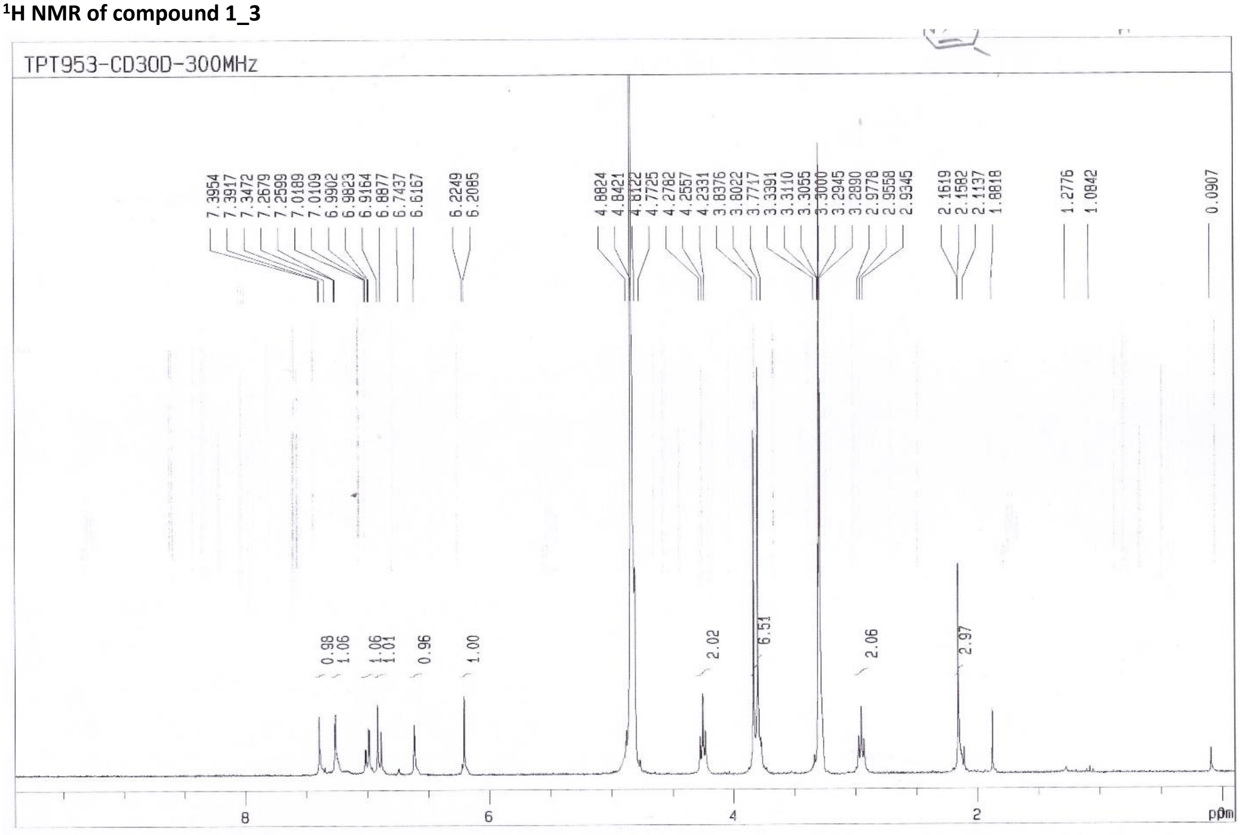

MS of compound 1_3

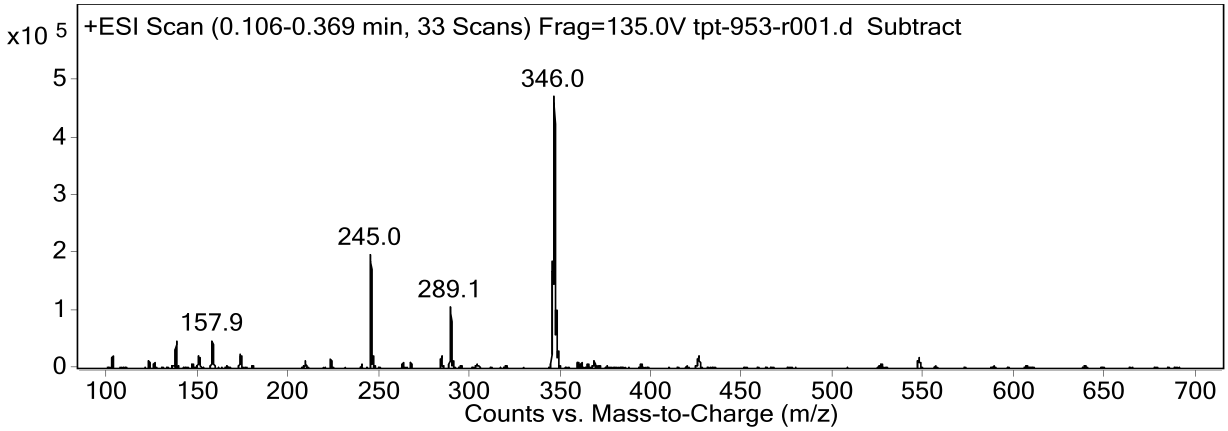

^{1}H NMR of compound 1_4

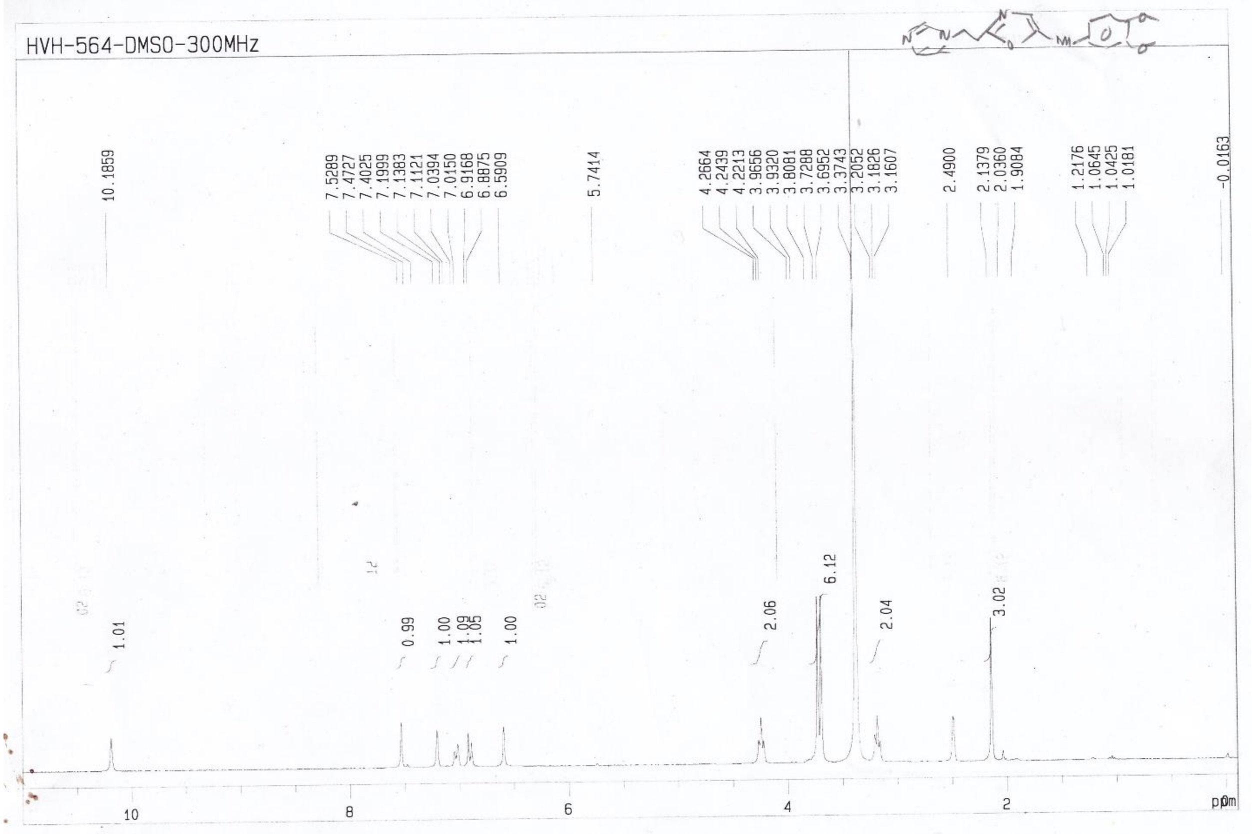

MS of compound 1_4

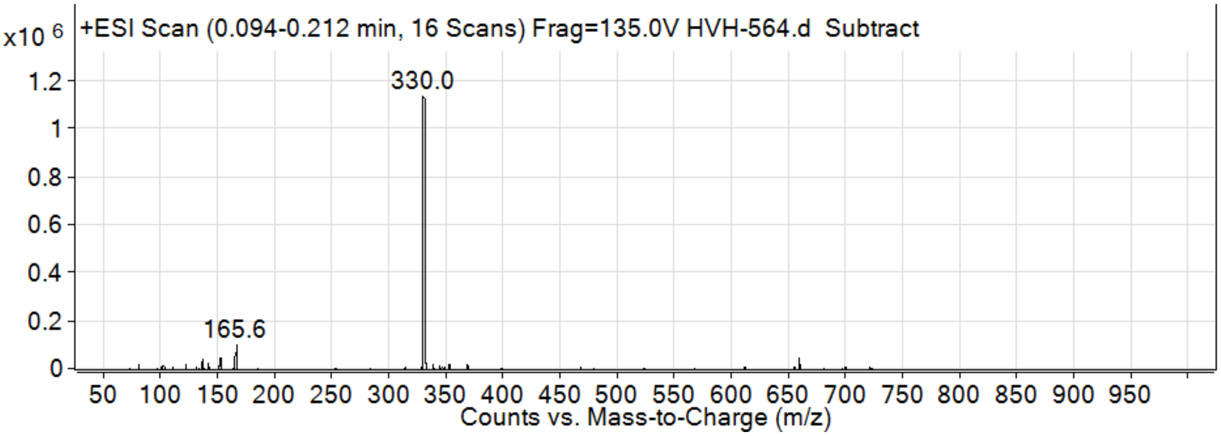

^{1}H NMR of compound 3_1

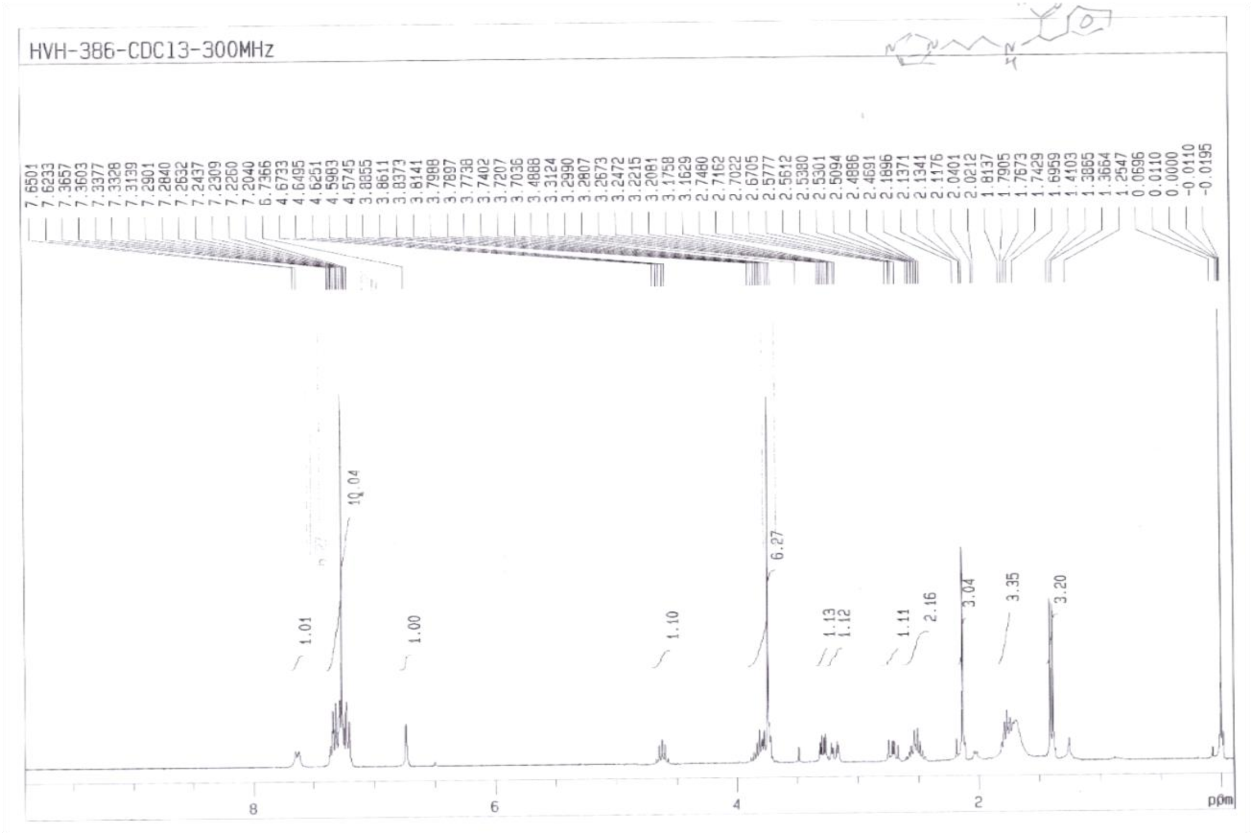

MS of compound 3_1

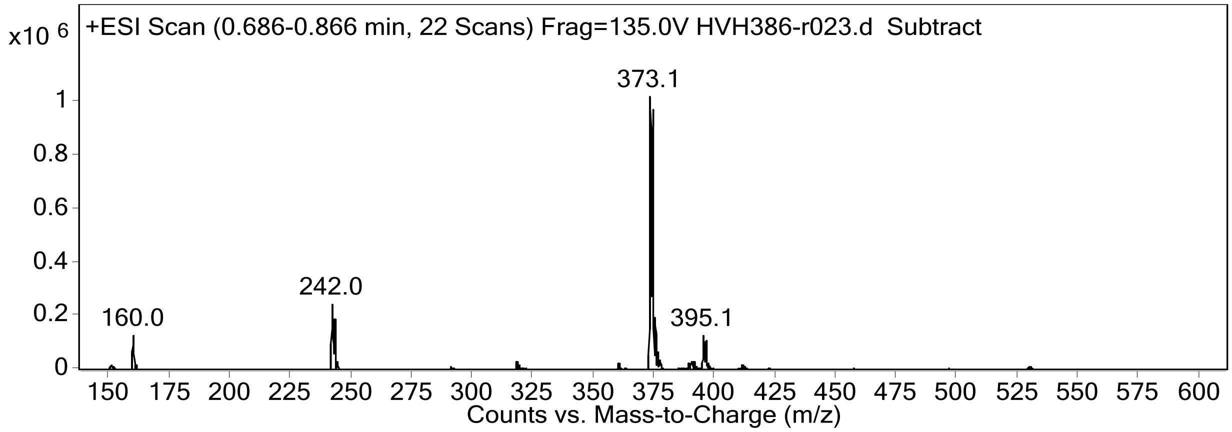

^{1}H NMR of compound 3_3

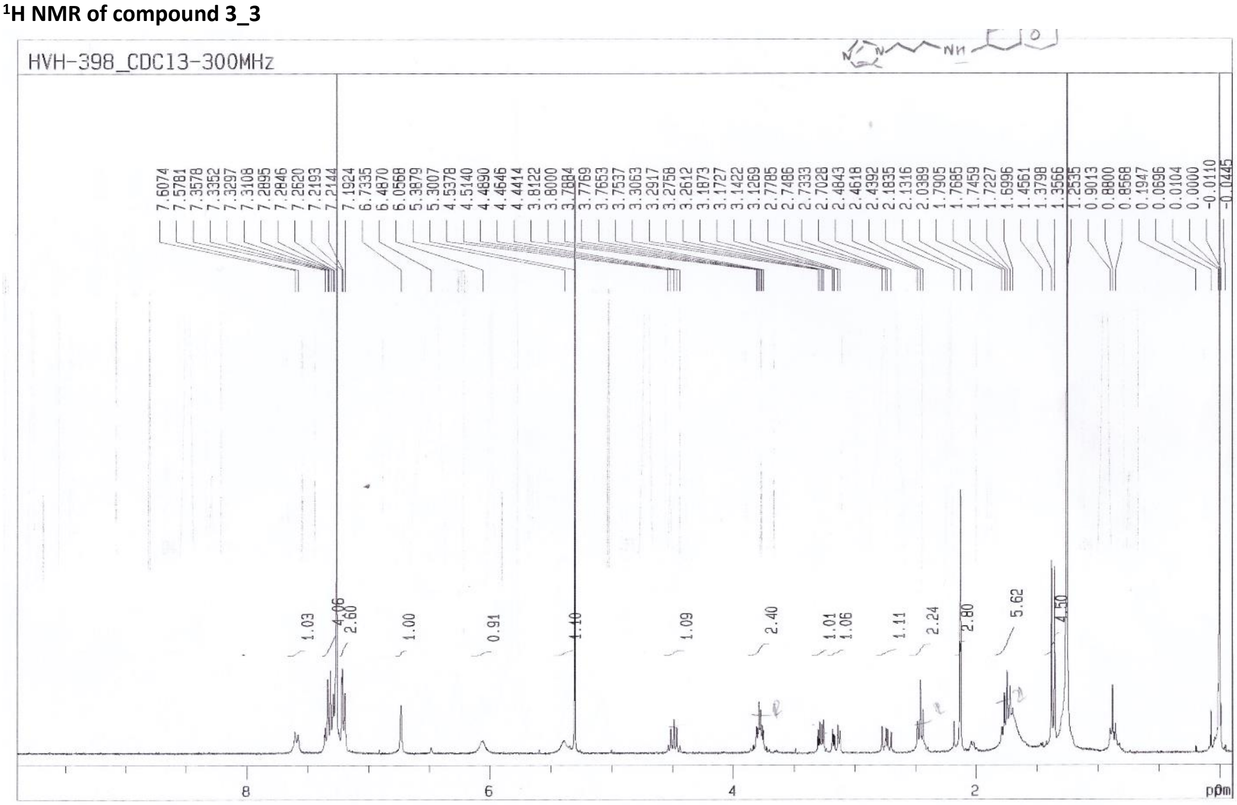

MS of compound 3_3

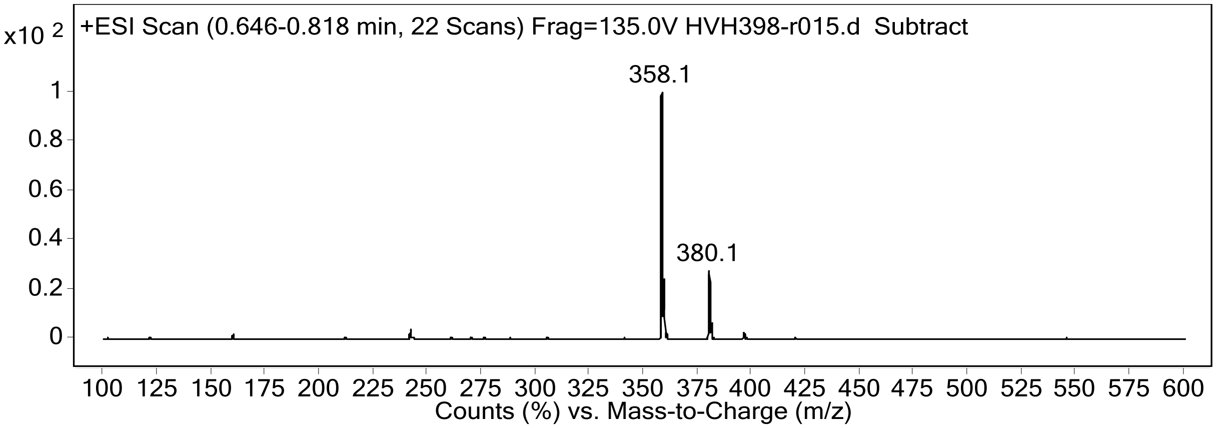

Reference

1. Buchholz M, Hamann A, Aust S, Brandt W, Böhme L, Hoffmann T, et al. Inhibitors for Human Glutaminyl Cyclase by Structure Based Design and Bioisosteric Replacement. J Med Chem. 2009;52(22):7069-80.
2. Trott O, Olson AJ. Improving the speed and accuracy of docking with a new scoring function, efficient optimization, and multithreading. J Comput Chem. 2010;31:455-61.
3. Morris GM, Huey R, Lindstrom W, Sanner MF, Belew RK, Goodsell DS, et al. AutoDock4 and AutoDockTools4: Automated docking with selective receptor flexibility. J Comput Chem. 2009;30(16):2785-91.
4. Ngo ST, Li MS. Top-leads from natural products for treatment of Alzheimer's disease: docking and molecular dynamics study. Mol Sim. 2013;39(4):279-91.
5. Ngo ST, Thu Phung HT, Vu KB, Vu VV. Atomistic investigation of an Iowa Amyloid-β trimer in aqueous solution. RSC Adv. 2018;8(73):41705-12.
